# Matching with time‐dependent treatments: A review and look forward

**DOI:** 10.1002/sim.8533

**Published:** 2020-04-03

**Authors:** Laine E. Thomas, Siyun Yang, Daniel Wojdyla, Douglas E. Schaubel

**Affiliations:** ^1^ Department of Biostatistics and Bioinformatics Duke University School of Medicine Durham North Carolina USA; ^2^ Duke Clinical Research Institute Duke University School of Medicine Durham North Carolina USA; ^3^ Department of Biostatistics, Epidemiology and Informatics, Perelman School of Medicine University of Pennsylvania Philadelphia Pennsylvania USA

**Keywords:** longitudinal matching, new‐user design, real‐world evidence, time‐dependent confounding, time‐varying treatment

## Abstract

Observational studies of treatment effects attempt to mimic a randomized experiment by balancing the covariate distribution in treated and control groups, thus removing biases related to measured confounders. Methods such as weighting, matching, and stratification, with or without a propensity score, are common in cross‐sectional data. When treatments are initiated over longitudinal follow‐up, a target pragmatic trial can be emulated using appropriate matching methods. The ideal experiment of interest is simple; patients would be enrolled sequentially, randomized to one or more treatments and followed subsequently. This tutorial defines a class of longitudinal matching methods that emulate this experiment and provides a review of existing variations, with guidance regarding study design, execution, and analysis. These principles are illustrated in application to the study of statins on cardiovascular outcomes in the Framingham Offspring cohort. We identify avenues for future research and highlight the relevance of this methodology to high‐quality comparative effectiveness studies in the era of big data.

## 1 INTRODUCTION

Sources of observational data are expanding rapidly and include electronic health records, insurance provider claims, and quality improvement registries. These resources provide an opportunity to generate real‐world evidence regarding the comparative effectiveness and safety of treatments. A common feature is that data are collected longitudinally. With respect to study follow‐up, treatments vary over time, as do potential confounders in treatment selection and consequences of treatment.

Causal inference from observational data requires special methodology when treatments are initiated over longitudinal follow‐up. A naive comparison of never treated versus ever treated patients (ie, defining the treatment indicator at time 0 based on future treatment experience) is subject to survival bias (immortal time bias).^1^, ^2^, ^3^, ^4^ An apparent solution is to include treatment as a time‐dependent covariate in a Cox model for time‐to‐event outcomes, possibly adjusting for other time‐dependent variables.^5^, ^6^, ^7^ However, this approach is not valid when time‐dependent confounders that impact treatment initiation are subsequently affected by treatment.^8^, ^9^, ^10^ Appropriate methods depend on the scientific purpose. Thus, it is helpful to distinguish two types of time‐varying treatment: (a) the treatment of interest is static but patients' treatment status may vary in the available observational data; and (b) the intervention of interest involves a strategy to vary treatment over time (time‐varying treatment strategy).^11^ In this tutorial, we are interested in first setting. The ideal experiment would randomize patients to one or more static treatments after diagnosis/onset or disease/infection, as do conventional randomized trials. In the available observational data, however, patients initiate treatment at different times. We review and discuss methods that match patients across longitudinal follow‐up, referred to subsequently as longitudinal matching (LM) methods.

As a motivating example, we study the efficacy of statins for prevention of cardiovascular outcomes in the Framingham Offspring Cohort. The Framingham Heart Study includes decades of follow‐up during which statin treatment initiation is observed, along with time‐dependent factors, such as low density lipoprotein (LDL) cholesterol, that are likely to confound the observational relationship between statin treatment and outcome. The relationship between LDL and statins involves both time‐dependent confounding, where LDL affects statins, but statins also affect subsequent LDL. This exemplifies the problem of a bidirectional relationship between time‐dependent confounders and time‐dependent treatment. Despite the complex longitudinal data, the experiment that we are interested in would randomize patients to statins vs no statins and follow them for subsequent outcomes. In this example, corresponding randomized studies are plentiful. Therefore, a relevant benchmark is available to which we can compare the results of LM. Meta‐analysis of clinical trials established a clear benefit of statins for cardiovascular outcomes (meta‐analytic risk ratio for major coronary events 0.76, 95% CI: 0.73‐0.79).^12^

The design and analysis of statin initiation in Framingham would ideally utilize best practices for matching over longitudinal follow‐up. Unfortunately, methods for this purpose are scattered across disciplines, characterized by unique jargon (Table 1) and comparisons are lacking. There is no single source of information to summarize the progress in this field and inform applied researchers. To remedy this, we conducted a thorough literature review. Details of this process are described below. Referring to a particular version, Schneeweiss et al (2011)^13^ conclude that the “balanced sequential cohort design may become a standard solution for working with secondary observational data that fit a broad range of comparative effectiveness and safety questions.” As the balanced sequential cohort design is one of many LM methods, we seek to connect similar methods so that researchers can take advantage of the substantial work in this area.

**Table 1:** Variations on longitudinal matching

Name	Reference	Contribution
*Statistics*:		
Balanced risk set matching^3^	Li et al, 2001^14^	Original concept and theory
	Haviland et al, 2007^17^	Group‐based trajectory models for longitudinal history
	Zubizarreta et al, 2014^24^	“Isolation” to reduce unmeasured confounding
Propensity score matching with time‐dep. covariates^3^	Lu, 2005^15^	Time‐dependent propensity score
Sequential stratification^2^	Schaubel et al, 2006^19^	Strong theory and feasibility in big data
	Schaubel et al, 2009^20^	Interaction with time‐dep. covariates, and IPCW for treatment switching
	Kennedy et al, 2010^22^	Methods comparison
	Taylor et al, 2014^23^	Simulation study and methods comparison
	Smith et al, 2015^25^	Use of a prognostic score matching and recurrent events
Sequential Cox models^1^	Gran et al, 2010^21^	Regression‐based approach with inverse probability of censoring weights for switching
Matching methods for …	Li et al, 2014^26^	Survival estimation and counterfactual theory
time‐dependent treatment	He et al (in press)^44^	Prognostic score matching
*Epidemiology*:		
Matched cohort design	Seeger et al, 2005^28^	Intuitive framework and worked example
Emulating a target trial^1^	Hernan et al, 2008^29^	Target trial concept with compelling example
	Danaei et al, 2013^4^	Worked example with clear rationale and detail
	Hernan et al, 2016^33^	Connection to big data
	Hernan et al, 2016^34^	Common failures in target trial emulation
Incident user cohort design^3^	Schneeweiss et al, 2010^30^	Design considerations in healthcare data
Balanced sequential cohort^3^	Schneeweiss et al, 2011^13^	Review of challenges in early marketing
Sequential matched cohort^3^	Gagne et al, 2012^31^	Semi‐automated safety monitoring
Inc. user cohort de.^3^	Rassen et al, 2012^32^	Adds high‐dimensional propensity score
Rolling entry matching^3^	Witman et al, 2019^36^	Similar to Lu (2005) with software
	Jones et al, 2017^35^	Software for rolling entry matching
*Economics*:		
None^3^	Sianesi, 2004^1^	Counterfactual theory with applied focus
Dynamic treatment	Fredriksson et al, 2008^37^	Counterfactual theory and survival estimation
matching^2,3^	Crepon et al, 2009^39^	Additional theory and example
	Vikstrom, 2017^40^	Variable selection and new estimands
Sequential causal models	Lechner, 2009^38^	Alternative approaches
*Medicine*:		
None^1,2^	Ray et al, 2002^41^	Intuitive approach, strong application with active control
Staggered cohort study	Blackburn et al, 2017^43^	Application in psychology with clear rationale

*Note*: Superscripts ^1,2, or 3^ correspond to approaches in Section 5.2.1, 5.2.2, and 5.2.3, respectively

This review and tutorial is organized as follows. First, we define a general class of LM methods (Section 2). In Section 3, we describe the process of literature review and findings. Based on the literature review, Section 4 outlines important considerations for study design. Alternative approaches to matching are described in Section 5. In Section 6 we review the analysis of outcome and interpretation. Section 7 illustrates the study of statins in Framingham with respect to design, matching and analysis. Decisions related to each of the preceding domains are explained. The results of three distinct LM methods are compared to the benchmark of clinical trial results. To the best of our knowledge, this is the first time alternative LM methods have been applied side‐by‐side to the same example. Similarities and differences are discussed. SAS code to replicate these analyses for a simulated example is available upon request. Finally, we offer concluding remarks and identify opportunities for future research (Section 8).

## 2 NOTATION AND DEFINITION

The common feature of LM methods is to mimic a randomized study, were randomization to have occurred at the observed treatment times. Methods in this class proceed as follows: begin with time scale, *s*, measured from a relevant time 0, such as first eligibility for treatment. Details on selection of time scales are considered in Section 4.2. Let *S*_*i*_ represent the possibly unobserved *start*‐time of treatment, and *D*_*i*_ and *C*_*i*_ denote death and censoring times, respectively, for patient *i* (*i*=1,…,*n*). Let *Y*_*i*_ represent the outcome of interest. For now, *Y*_*i*_ may be continuous, binary, or time‐to‐event, possibly equal to *D*_*i*_ when the outcome of interest is death. Patients are considered at‐risk if they are alive and under follow‐up, indicated by *R*_*i*_(*s*)=*I*(*D*_*i*_∧*C*_*i*_≥*s*) where *a*∧*b* denotes the minimum of *a* and *b*. The covariate vector of features measured at time *s* is denoted ***Z***_*i*_(*s*), and the full covariate history prior to time *s* is $Z_{i}^{*}(s)=\{Z_{i}(x);0\leq x\leq s\}$. To incorporate the possibility that patients can become ineligible for treatment (develop a contraindication, or recover), ${ℰ}_{i}(s)$ takes the value of 1 when a patient is eligible and 0 when the patient is ineligible. In some cases, this would be 1 for all patients over all time.

**Figure 1:**
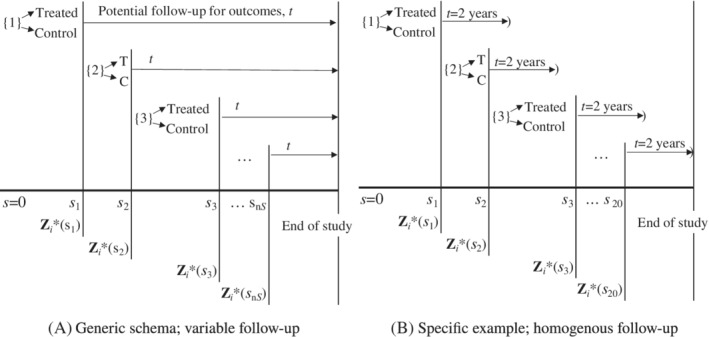
Schema of enrollment across *j*=1,…,*n*_*S*_ longitudinal pseudo‐experiments. Time scale, s, represents time since first eligibility for treatment initiation. {*j*} represents the set of eligible patients at time *s*_*j*_ satisfying *R*_*i*_(*s*_*j*_)=1, ${ℰ}_{i}(s_{j})=1$, and *S*_*i*_≥*s*_*j*_ for *i*=1,…,*n* patients. Any patients with *S*_*i*_=*s*_*j*_ are treated and the remainder with *S*_*i*_>*s*_*j*_ are controls. Covariate history $Z_{i}^{*}(s_{j})$ is available to the *j*th experiment. A, Generic scheme; variable follow‐up. B, Specific example; homogenous follow‐up

A pseudo‐experiment is initiated each time a subject is observed to receive treatment. The ordered, observed treatment times are denoted *s*_*j*_ for *j*=1,…,*n*_*S*_, where *n*_*S*_ is the total number of unique times at which treatment is started. In the simplest form, treated patients are “matched” to *all* available controls *only* according to eligibility and risk status. All patients are entered into the *j*th pseudo‐experiment if they remain at risk, *R*_*i*_(*s*_*j*_)=1, eligible, ${ℰ}_{i}(s_{j})=1$, and they were not treated previously, *S*_*i*_≥*s*_*j*_ (Figure 1A). In the special case where initiation times, *s*_*j*_, are measured very precisely, there will only be one individual who is treated in the *j*th pseudo‐experiment and the rest are potential controls. Generally, when time is measured more coarsely, multiple patients may initiate at the same time and the *j*th experiment will have a group of treated patients and a corresponding group of controls (Figure 1A). Importantly, this basic LM framework does not prescribe how to select controls and address imbalances in the covariates ***Z*** ^*^(*s*_*j*_), which are likely present in observational data. Various LM methods differ in the approach to adjustment for ***Z*** ^*^(*s*_*j*_) and corresponding model for outcome (see Section 5). What all LM methods have in common is the creation of pseudo‐experiments (not always explicit) for which ***Z*** ^*^(*s*_*j*_) is known when the experiment is initiated, at “baseline” *s*_*j*_. Therefore, this information is available to be used in a variety of ways, analogous to cross sectional data.

Within each pseudo‐experiment, let *t* denote the follow‐up time for outcomes that accrues after initiation of the respective experiment. For each individual in the *j*th experiment, *t*=0 coincides with *s*=*s*_*j*_. When *Y*_*i*_ is a time‐to‐event outcome, the follow‐up might look like Figure 1A. When *Y*_*i*_ is continuous or binary, the follow‐up is generally restricted to a common time point like Figure 1B. Survival endpoints are emphasized in this article due to relative prominence in the literature and in comparative effectiveness analyses. However, the same LM methods can be paired with continuous^14^, ^15^ and binary outcomes.^16^, ^17^

## 3 LITERATURE REVIEW

We undertook a literature review to identify LM methods consistent with the framework above. This included both a systematic search in the Web of Science and extensive cross‐referencing. The search was conducted in two phases. A preliminary ad hoc search yielded 33 publications with at least some relevance to LM. From these, we identified the following key words: time‐dependent covariates, time‐dependent confounding, balanced risk set matching, sequential stratification matching, sequential Cox models, propensity score matching with time‐dependent covariates, matching methods for longitudinal data, comparative effectiveness studies in longitudinal data, comparative effectiveness studies in Medicare claims data, time‐varying exposure, and dynamic matching. A second search based on these key words yielded 239 potentially important papers. Among these, 56 remained after screening for relevance (unique from the original 33). Cross‐referencing and consultation with collaborators helped to identify another 47 related articles. Finally, 136 articles were carefully reviewed. We identified articles that met the following criteria: (a) proposes or advances LM as described in Section 2; (b) includes a strong rationale or theoretical justification; and (c) choices are explained and sufficient details are provided to facilitate appropriate application of the methodology. After applying these criteria, 30 publications are the focus of this review (Table 1), although others that address related topics are referenced subsequently. In Table 1, there are 14 distinct LM methods, as distinguished by having different names and having been conceptualized as a distinct approach. Publications that intentionally build upon an initial method are grouped together under the same name.

The origin of LM traces back to Prentice et al (1978) who proposed matching patients at a common time point during longitudinal follow‐up.^18^ Since then, adaptations and similar approaches have been developed in statistics,^3^, ^14^, ^15^, ^19^, ^20^, ^21^, ^22^, ^23^, ^24^, ^25^, ^26^ psychology,^16^, ^17^ criminology,^27^ epidemiology,^4^, ^13^, ^28^, ^29^, ^30^, ^31^, ^32^, ^33^, ^34^, ^35^, ^36^ economics,^1^, ^37^, ^38^, ^39^, ^40^ and medicine.^41^, ^42^, ^43^ In the statistical literature, the earliest LM method was balanced risk set matching,^14^ although the conceptualization of a series of pseudo‐experiments was formalized by sequential stratification.^19^ A similar concept of emulating a target trial through a sequence of nonrandomized trials appears to have arisen independently in epidemiology^29^ and was framed as a dynamic treatment problem in economics.^37^ All methods proposed in the statistical and economics literature include at least some theory to establish a causal interpretation, using potential outcomes or causal models to define assumptions. Corresponding methods in epidemiology and medicine tend to emphasize well accepted principles of study design.

From this literature, we identified important domains to consider during study design, matching, and analysis of outcomes. Details are provided in Sections 4, 5, and 6 and summarized in Table 2.

**Table 2:** Key steps and decisions in longitudinal matching

	Section
Design	4
Target trial specification helps to guide analytic decisions	4.1
Multiple time scales are important and the primary scale should be clinically meaningful	4.2
Use LM methods to facilitate a new‐user design. Use a wash‐out period as needed.	4.3
Select active vs inactive controls to align with the target trial	4.4
Eligibility can be required for entry into an experiment but is not relevant afterwards	4.5
Treatment switching; specify which are acceptable and which depart from the question of interest	4.6
Matching	5
Select confounders that best aggregate prognostic information	5.1
Longitudinal matching methods	5.2
‐ Option 1: Match on time and eligibility, parametric regression models address confounding	5.2.1
‐ Option 2: Exact matching on important strata, plus parametric regression models	5.2.2
‐ Option 3: Matching on all relevant confounders, consider propensity and prognostic scores	5.2.3
Recent recommendations for matching with cross sectional data are relevant	5.3
Assessment of balance is important when relying on covariate matching (Option 3)	5.4
Analysis of outcome	6
LM methods are typically conditional on covariates, rather than marginal	6.1
Describe treatment initiation times, and subsequent follow‐up to inform generalizability	6.2
Censoring may introduce bias; consider weighting (IPCW)	6.3
Effect modification by time‐varying factors; facilitated by Options 1 and 2	6.4
Variance estimation should account for correlation induced by repeated data	6.5

## 4 STUDY DESIGN

### 4.1 Target trial specification

A starting point for LM is the specification of the target trial.^19^, ^29^, ^33^, ^45^ Target trial emulation has been the primary framework for a series of analyses in epidemiology,^29^, ^33^ but was also the foundation for sequential stratification.^19^ Decisions about design, methods, and analysis can be guided by first defining the interventional study of interest. The target trial does not have to be feasible. For example, Schaubel et al (2006) conceptualize the randomization of patients on the wait‐list for kidney transplant. This will never occur due to logistical and ethical considerations. However, it is helpful to establish how such a study would proceed. Who would be eligible? How long would the follow‐up accrue? What subsequent interventions would be allowed? The target trial can also be highly pragmatic. For example, we may be interested in the randomization to an initial therapy, after which patients behave as they do in the real world, with heterogeneous adherence or dosing practice. The value of target trial specification is to clarify what is and is not being attempted with observational data so that decisions are aligned with that purpose.

### 4.2 Time scales

Examples of LM often measure treatment initiation times from the occurrence of a critical event such as time since human immunodeficiency virus (HIV) infection,^21^ time since surgery,^46^ and time since first‐line therapy.^22^ In these examples, time *s*=0 is the first time a patient becomes eligible for the therapy of interest, and *s*_1_ through $s_{n_{S}}$ represent treatment initiation times since first eligibility. Others use calendar time as the scale over which pseudo‐experiments are defined.^28^, ^47^ A graphical comparison of different times scales is available in Supplementary Material. Related work has emphasized the need to consider multiple time scales, including calendar time, age since birth, time since an index event, and time since study start.^3^, ^48^ LM methods can account for all of these time scales, simultaneously, by using one as the time scale for matching (*s*) and incorporating the rest into $Z_{i}^{*}(s)$. The time scale selected for matching should be clinically relevant or create strata in which subjects would be similar with respect to prognosis.^22^ Consequently, the ideal scale may often be one that is highly related to outcome.^19^ Whichever factor is used as the time scale for matching will be handled in a nonparametric way avoiding assumptions like proportional hazards.^48^

A time scale that is highly correlated with treatment should be considered carefully. It may reflect a natural experiment, or rapid shift in treatment utilization, where very little else has changed. For example, calendar time may be highly correlated with treatment initiation if the study period includes time before and after regulatory approval. If the time period is otherwise relatively short, calendar time may have little reason to affect outcomes other than the accessibility of treatment. That is, time period behaves like an instrumental variable (causing treatment assignment, but otherwise not causing outcome). Adjustment for an instrumental variable can increase bias^49^, ^50^ and instrumental variables should not be included as the time scale, nor a covariate.^48^

A final consideration regarding time‐scale is coarsening. The process of LM can be computationally demanding if there is a large number of patients who initiate treatment, and time is measured continuously. While many examples match individuals on a daily time scale,^19^, ^20^, ^51^ others are monthly,^1^, ^21^, ^39^ quarterly,^13^ yearly,^16^ and bi‐annual.^29^ Smaller intervals are better for capturing the most recent information prior to treatment initiation and events occurring shortly after treatment initiation.

### 4.3 New user design

LM methods facilitate the creation of a new user comparison from observational data, particularly when the control group is inactive (ie, untreated standard of care). In brief, new users are those who have just initiated treatment (or placebo in an experiment), whereas prevalent users have prior exposure (prior to the study period). A new user design captures information on pre‐treatment characteristics and begins follow‐up at the time of treatment initiation, just as an interventional study would. Prior users are typically excluded. There are well‐known advantages to studying treatments from inception.^13^, ^30^, ^33^, ^34^, ^45^ This facilitates appropriate adjustment for confounding because pre‐treatment covariates rather than post‐treatment covariates should be used for statistical adjustment. With respect to the *j*th experiment (LM), it is clear that covariate information prior to treatment initiation, $Z_{i}^{*}(s)$ where *s*≤*s*_*j*_, is relevant to adjustment (Figure 1), whereas post‐initiation, $Z_{i}^{*}(s)$ and *s*>*s*_*j*_, is not.^3^, ^16^, ^30^, ^45^ Moreover, follow‐up for outcomes begins at the time of treatment initiation (and corresponding time for matched controls). This ensures that all outcomes are captured, as they would be in a clinical trial, starting from treatment initiation (or a corresponding time without treatment). In sources such as Medicare claims data and electronic medical records, it is often necessary to specify a wash‐out period (ie, first 1‐2 years of available data) in order to identify new users. Patients receiving treatment during the wash‐out period are considered prevalent users and excluded from eligibility. After the wash‐out period, occurrence of treatment is assumed to be a new initiation.^4^ Historically it has been difficult to study new users due to limited sample size. The increasing availability of big data sources presents an opportunity to improve study design.^33^ All of the reviewed LM methods correspond to a new user design (assuming a wash‐out period has been applied where needed) and pseudo‐experiments correspond to treatment initiation times (Figure 1).

### 4.4 Active versus inactive controls

The basic LM framework described in Section 2 describes a pool of potential controls for each patient who initiates treatment in the *j*th experiment. Frequently, there are no additional criteria. That is, the controls represent a pseudo‐placebo where the goal is to compare a single active treatment to remaining untreated (otherwise treated by the standard of care), the ideal experiment would involve a placebo.^4^ Similar methods can be used to create pseudo‐experiments in which both comparator arms initiate an active treatment at or approximately around time *s*.^13^ There are potential advantages to comparing active treatments.^13^, ^24^, ^30^, ^52^ “Using such active comparators may mitigate bias because initiators of the treatment of interest and the active comparator are expected to have a similar health status and use of the health care system and comparable quality of information.”^52^ When the comparison of active treatments is of substantive interest, the analysis may benefit from these advantages. Alternatively, when the ideal experiment would include a placebo, investigators can attempt to identify an active comparator that is expected to behave like a placebo with respect to the outcome of interest. This decision involves compromises: (a) whether an active comparator behaves like a placebo is not guaranteed; and (b) the population to which treatment effect estimates are generalizable may be limited by the characteristics of patients receiving active control.^24^, ^52^ Investigators should carefully consider the likely benefits versus limitations in a particular context.

### 4.5 Eligibility

There are at least two kinds of eligibility that are relevant to the study of treatments that are initiated longitudinally. The most obvious involves absolute contra‐indications to treatment; factors that would make a patient ineligible to receive an intervention of interest. For example, Schaubel et al (2009) sought to evaluate the benefit of liver transplantation in patients wait‐listed for a transplant due to end stage liver disease.^20^ Patients could be removed from the wait‐list due to recovery of liver function or because they are too sick to undergo surgery. Either condition renders an individual ineligible for the population of wait‐listed patients. A patient who is too sick to undergo transplant at time *s*_3_ will have ${ℰ}_{i}(s_{3})=0$, but may recover and be eligible at time *s*_5_ such that ${ℰ}_{i}(s_{5})=1$. Only eligible patients, ${ℰ}_{i}(s_{j})=1$, are allowed to enter the *j*th experiment. However, once a patient has entered an experiment (treated or untreated), future changes in eligibility are part of the outcome process and, generally, do not result in censoring or exclusion.^3^, ^16^, ^30^ One exception, discussed in Section 4.6, is cross‐over or treatment switching.

The existence of a large convenience sample does not imply that everyone in the data set is of interest at all times. In a study of survival outcomes in bariatric surgery patients, for example, Aterburn et al (2015)^47^ were only interested in a population of severely obese individuals, defined by body mass index (BMI) greater than 40, from among a large Veterans affairs (VA) database. With longitudinal data available from 2000‐2011, the obesity status of patients varied over time, as did eligibility for the study. In a setting like this, the indicator ${ℰ}_{i}(s_{j})$ can capture time‐varying inclusion criteria.

### 4.6 Treatment switching

The creation of pseudo‐experiments does not guarantee that subjects will adhere to their initial treatment status. During longitudinal follow‐up, some patients who were initially controls may start treatment and treated patients may stop. This is analogous to randomized trials. The intent‐to‐treat (ITT) analysis will allow these switches to occur without changing follow‐up for outcomes. In various settings, the ITT effect is of primary interest because future switching will remain an option and is itself an important part of the outcome.^1^, ^16^, ^19^, ^28^ To support interpretation of the ITT, Ray et al (2012) provide a description of treatment switching patterns across 18 pseudo‐experiments.^42^ Similarly, Schaubel et al (2006) evaluated the choice to accept a kidney transplant from an expanded criterion donor (ECD) for patients with end‐stage renal disease, who could alternatively wait for a non‐ECD kidney that would have a lower probability of transplant failure.^19^ They note that the relevant question is not a comparison of ECD and non‐ECD kidneys, but rather “Would I be better off accepting an ECD organ, given that, if I do not accept it, I could subsequently be offered a non‐ECD organ?.” Pseudo‐experiments are created and patients are not censored if they subsequently receive a transplant (ECD or non‐ECD) nor if they are subsequently removed from the waitlist. All of these downstream treatment changes are regarded as part of the standard of care. The ideal analysis is ITT, including all changes that happen naturally after treatment initiation.

As with randomized experiments, the ITT interpretation is sometimes less interesting than a hypothetical scenario in which everyone had remained on their initial treatment, throughout follow‐up. This is often called the per‐protocol treatment effect, interpreted as “the effect of starting and adhering to the treatment assignment.” During the design stage, it is important to determine which of these treatment effects is of interest. Specification of the target trial can help to guide this decision. What reasons for switching would be acceptable (to remain under follow‐up) and which violate the intended treatment strategy? Methods to estimate a per‐protocol effect are described in Section 6.3 and are widely used along with LM.

## 5 MATCHING

A major difference between LM methods is the handling of the covariate history, $Z_{i}^{*}(s_{j})$, between patients who initiate treatment and controls. Broadly speaking, there are three approaches: (a) methods that match only on longitudinal eligibility (as in Section 2) and depend on parametric models to adjust for confounding by $Z_{i}^{*}(s_{j})$;^21^, ^29^, ^42^ (b) methods that incorporate components of $Z_{i}^{*}(s_{j})$ into matching, but add parametric models to adjust for the remaining differences;^19^, ^20^, ^22^, ^25^ and (c) methods that fully adjust for $Z_{i}^{*}(s_{j})$ during matching.^1^, ^14^, ^15^, ^16^, ^24^, ^27^, ^28^, ^30^, ^39^, ^51^, ^53^ These are described in Sections 5.2.1, 5.2.2, and 5.2.3, respectively. Keeping these options in mind, we first consider what to include among covariates ***Z*** ^*^(*s*).

### 5.1 Selection of confounders

All of the methods considered here rely on the assumption of no unmeasured confounding; the study must measure and account for time‐dependent confounders that induce one individual to receive treatment at time *s*_*j*_ and another individual not to. As with cross‐sectional data, confounders are common causes of treatment selection and subsequent outcomes.^54^ A particularly strong case can be made for no‐unmeasured confounding when a measure of the longitudinal outcome process can be obtained prior to treatment initiation. Haviland et al (2007) note that a “common mistake in studying people over time is to designate certain variables as predictors and others as outcomes,” such that important variables on which to adjust may be missed because they have been designated as outcomes.^16^ LM provides a solution by “keeping time in order” so that everything measured before a pseudo‐experiment may be incorporated into $Z_{i}^{*}(s_{j})$, including prior measurements of a variable that will define the outcome at a later date.^3^, ^16^, ^23^ For example, Taylor et al (2014) modeled longitudinal prostate specific antigen (PSA) with mixed models, and obtained best linear unbiased predictors of log(PSA) and the slope of log(PSA), evaluated at each *s*_*j*_.^23^ These were incorporated into the matching process so that patients who initiated salvage androgen deprivation therapy (SADT) for prostate cancer would be similar to their assigned controls on the pre‐treatment trajectory of PSA, a known determinant of cancer recurrence and a known mechanism for deciding when to initiate SADT. Nieuwbeerta et al (2009) took this a step further by combining group‐based trajectory models with LM. Group‐based trajectory models can be used to identify groups of individuals who appear to be on a similar developmental pathway. Therefore, matching, using propensity scores, was conducted within each trajectory group.

The preceding examples demonstrate unique approaches to covariate adjustment, which are possible in longitudinal data. As with cross‐sectional studies, covariates are identified by clinical knowledge, considering the well‐known factors that guide treatment decisions and effect outcome, rather than statistical significance or automatic variable selection.^15^ Statistical significance and predictive metrics, such as the C‐index, are not appropriate ways to assess the success of confounding adjustment and that is not different in the longitudinal setting.^55^ However, high‐dimensional propensity score algorithms have recently been proposed to identify potential confounders from large healthcare databases^32^, ^56^ and applied to create longitudinally matched samples.^32^ To the extent, these methods capture information that would otherwise be unmeasured they may reduce confounding.

Another consideration is that covariates may be measured on a different schedule than treatment initiation times. Some components of ***Z***_*i*_ may not be known at time *s*_*j*_, but last measured at some *s*<*s*_*j*_. This is generally handled in one of the two ways. The last observation can be carried forward to time *s*_*j*_. This is reasonable if important covariates are measured often enough that they would remain relatively stable between measurements or if changes would tend to induce symptoms, which would, in turn, accelerate measurements.^20^, ^21^ On the other hand, patient monitoring is not perfect and longitudinal covariates can also be updated by linear interpolation.^20^, ^23^, ^57^

### 5.2 Methods

#### 5.2.1 Matching only on time and eligibility

The simplest LM method creates pseudo‐experiments based on time and eligibility as in Section 2 and Figure 1.^21^, ^29^ See Supplementary Material for illustration with individual patients. For each *s*_*j*_, there is an experiment with case(s) and controls for whom the longitudinal covariate history, ***Z*** ^*^(*s*_*j*_), is known. To estimate a treatment effect, Gran et al (2010) posit a Cox proportional hazards model for outcome that includes this history ***Z*** ^*^(*s*_*j*_) and a treatment indicator. Suppose time is coarsened and numerous patients initiate at time *s*_*j*_, so that we can imagine fitting a model for outcomes in the *j*th pseudo‐experiment alone. The hazard for individual *i* in pseudo‐experiment *j* is given by:

(1) $$\begin{array}{ll} \lambda_{i(j)}(t;s_{j}|\theta ,\beta_{j})=\lambda_{0(j)}(t;s_{j})\exp\{\theta^{\prime }Z_{i}(s_{j})+\beta_{j}I(S_{i}=s_{j})\}, & \end{array}$$

where λ_0(*j*)_(*t*;*s*_*j*_) is the baseline hazard at *t* time units following time *s*_*j*_, $\lambda_{0(j)}(t;s_{j})=\lim_{\delta \to 0}\delta^{-1}P\{t\leq Y_{i}<t+\delta |Y_{i}\geq t,S_{i}>s_{j},R_{i}(s_{j})=1,{ℰ}_{i}(s_{j})=1,Z_{i}(s_{j})=0\}$. Therefore λ_*i*(*j*)_(*t*;*s*_*j*_|**θ**,β_*j*_) is the hazard function corresponding to the random variable *Y*_*i*_ conditional on $\left[S_{i}\geq s_{j},R_{i}(s_{j})=1,{ℰ}_{i}(s_{j})=1,Z_{i}(s_{j})\right]$. For notational simplicity, the hazard depends on ***Z*** ^*^(*s*_*j*_) only through ***Z***(*s*_*j*_), though the model should include any relevant history ***Z*** ^*^(*s*_*j*_) based on substantive knowledge. Cox regression on the outcomes with respect to time *t*, for patients in the *j*th pseudo‐experiment, is used to estimate the parameter β_*j*_, which is of primary interest. The information across *n*_*S*_ pseudo‐experiments may be combined to estimate an overall treatment effect by fitting a stratified Cox model, stratifying on the index *j*. Gran et al (2010) describe assumptions for this to estimate a causal treatment effect. That is, there are no unmeasured confounders, the model for estimating the hazard rate is correct, and β_*j*_ is constant across strata. The last assumption can be relaxed by viewing the pooled estimate as a weighted average of stratum‐specific causal effects.

#### 5.2.2 Stratification with exact matching

Stratification on important components of ***Z*** provides a compromise where factors that are hard to model, or strongly related to outcome may have their own, unspecified baseline hazard. Similar to Schaubel et al (2006), define stratum, *k*, based on a select set of covariates. Suppose age (in years) and geographic U.S. state are selected for stratification. The range of age spans 18‐88. The combinations of age (70 levels) and state (50 levels) yield 3500 strata. For notational simplicity, we assume that treatment initiation times, *j*=1,…,*n*_*S*_, are unique or can be ordered so that *n*_*S*_ is the total number of patients who initiate treatment (See Supplementary Material for alternative notation and illustration with individual patients). Let *k*_*i*_(*s*_*j*_) denote the stratum membership at the time of the *j*th experiment for the *i* ^*th*^ patient, and ***Z***_*i*_(*s*_*j*_) includes the remaining covariates. The algorithm is Section 2 is modified. Define the index patient, *j*, as the one receiving the *j*th treatment initiation. All patients are entered into the *j*th experiment as controls if they remain at risk, *R*_*i*_(*s*_*j*_)=1, eligible, ${ℰ}_{i}(s_{j})=1$, they were not treated in a previous experiment, *S*_*i*_≥*s*_*j*_, *and* if they are contemporaneously in the same stratum as patient *j*, *k*_*i*_(*s*_*j*_)=*k*_*j*_(*s*_*j*_). Thus, the index patient is matched exactly to all eligible controls based on the combinations of covariates used to define strata.

For a time‐to‐event outcome, *D*_*i*_, the model for the hazard looks the same as before:

(2) $$\begin{array}{ll} \lambda_{i(j)}(t;s_{j}|\theta ,\beta )=\lambda_{0(j)}(t;s_{j})\exp\{\theta^{\prime }Z_{i}(s_{j})+\beta I(S_{i}=s_{j})\}, & \end{array}$$

where λ_0(*j*)_(*t*;*s*_*j*_) is the baseline hazard at *t* time units following time *s*_*j*_ for the *j*th matched stratum. Compared with Equation (1), Equation (2) applies to a more limited subgroup of patients, specifically those with *k*_*i*_(*s*_*j*_) equal to *k*_*j*_(*s*_*j*_) and $\lambda_{0(j)}(t;s_{j})=\lim_{\delta \to 0}\delta^{-1}P\{t\leq Y_{i}<t+\delta |Y_{i}\geq t,S_{i}>s_{j},R_{i}(s_{j})=1,{ℰ}_{i}(s_{j})=1,Z_{i}(s_{j})=0,k_{i}(s_{j})=k_{j}(s_{j})\}$. The information across *n*_*S*_ strata is combined to estimate an overall treatment effect by fitting a stratified Cox model, stratifying on the index *j*.^19^, ^20^, ^22^, ^23^

This approach is appealing when it makes sense to view patients as comparable only within a common strata. The assumptions for a causal interpretation are essentially the same as in Gran et al (2010); however, the model defined by Equation (2) makes parametric assumptions on fewer covariates. This method is also practically useful with large healthcare databases when the number potential controls is enormous. A preliminary search, requiring perfect agreement on a few strata variables, is computationally feasible, whereas consideration of the full covariate vector may be easier once data have been collapsed into experimental strata.^47^

#### 5.2.3 Covariate matching

In many settings, it may be hard to determine which variables should be viewed as stratum, *k*, versus covariates, ***Z***. Balanced risk set matching,^14^ addressed imbalances between treatment groups, on all covariates ***Z***, directly in the matching process. They recommended optimal balance matching based on a Mahalanobis distance,^14^ which is available in the OPTMATCH R package^58^ and widely adopted,^15^, ^16^, ^51^ though others subsequently used nearest neighbor matching,^53^ and caliper matching.^27^ As with cross‐sectional studies, it is appealing to match on a time‐dependent score (eg, propensity score, prognostic score, Euclidean distance), rather than the vector of covariates ***Z***_*i*_(*s*_*j*_) explicitly.^1^, ^15^, ^39^

The use of a score implies two phases of analysis. Lu (2005) estimated a propensity score based on a Cox model for the hazard of receiving treatment,^15^ based on time‐varying covariates,

(3) $$\begin{array}{ll} \lambda_{i}^{T}(s|\gamma )=\lambda_{0}^{T}(s)\exp\{\gamma^{\prime }Z_{i}(s)\}, & \end{array}$$

where $\lambda_{i}^{T}(s|\gamma )$ is the hazard of starting treatment for patient *i* over time scale *s*, and ***Z***_*i*_(*s*) contains the value of covariates ***Z***_*i*_, which are potentially time‐varying and updated to their value at time *s*. Using standard software for Cox proportional hazard regression with time‐dependent covariates, the propensity is estimated for each patient (on the log hazard scale) taking the value ${\hat{\gamma }}^{\prime }Z_{i}(s_{j})$ at the initiation of the *j*th experiment. Controls are selected from among those who remain at risk, *R*_*i*_(*s*_*j*_)=1, eligible, ${ℰ}_{i}(s_{j})=1$ and were not treated in a previous experiment, *S*_*i*_≥*s*_*j*_, by minimizing the distance function $\delta ={\left\{{\hat{\gamma }}^{\prime }[Z_{i}(s_{j})-Z_{j}(s_{j})]\right\}}^{2}$. Within each strata (*j*=1,…,*n*_*S*_), matching proceeds as with a cross‐sectional study. Other time‐dependent propensity matching methods have been proposed, allowing more flexible models for the time‐dependent propensity score,^1^, ^28^, ^39^ or matching on a prognostic score rather than a propensity score.^25^, ^44^

Once the matched sets are created, the analysis proceeds with standard methods for paired data. Lu (2005) derived paired differences in a continuous outcome of pain score measured at 3 months post‐baseline. They used a Wilcoxon signed rank test to evaluate the null hypothesis of no treatment effect. For a time‐to‐event outcome, *D*_*i*_, a Cox proportional hazards model for the *j*th pair is

(4) $$\begin{array}{ll} \lambda_{i(j)}(t;s_{j}|\beta )=\lambda_{0(j)}(t;s_{j})\exp\{\beta I(S_{i}=s_{j})\}, & \end{array}$$

where $\lambda_{0(j)}(t;s_{j})=\lim_{\delta \to 0}\delta^{-1}P[t\leq Y_{i}<t+\delta |Y_{i}\geq t,S_{i}>s_{j},R_{i}(s_{j})=1,{ℰ}_{i}(s_{j})=1,pair=j]$. A pooled estimate of β may be obtained by combining the pseudo‐experiments into a single model, stratified on the pair *j*. Alternatively, the covariates used in matching could be included in the regression model, yielding

(5) $$\begin{array}{ll} \lambda_{i(j)}(t;s_{j}|\theta ,\beta )=\lambda_{0(j)}(t;s_{j})\exp\{\theta^{\prime }Z_{i}(s_{j})+\beta I(S_{i}=s_{j})\}, & \end{array}$$

where $\lambda_{0(j)}(t;s_{j})=\lim_{\delta \to 0}\delta^{-1}P[t\leq Y_{i}<t+\delta |Y_{i}\geq t,S_{i}>s_{j},R_{i}(s_{j})=1,{ℰ}_{i}(s_{j})=1,Z_{i}(s_{j})=0,\text{pair}=j]$.^23^ This approach is similar to “doubly robust” methods and often preferred in the time‐invariant setting for providing additional adjustment where matches are not perfect.^59^ On the other hand, it requires parametric modeling of the relationship between covariates ***Z***_*i*_(*s*_*j*_) and outcome.

#### 5.2.4 Matching algorithm

The methods described in Sections 5.2.1 and 5.2.2 create coarsely matched groups that are expected to be comparable in important ways, such as having a common baseline hazard. The concept of matching “with replacement” is relevant as the same individual can be a control for multiple treated patients. Although patients who initiate an experiment are removed from later risk sets (ie, eligibility for the *j*th experiment requires *S*_*i*_≥*s*_*j*_), controls in a given experiment remain eligible for future experiments, where they may serve as controls again or may also initiate an experiment if they become treated. The resulting strata are not independent and these must be accounted for in variance estimation (Section 6.5).

In Section 5.2.3, methods are described that create pairs at the individual level, for every treated patient. A recent review of matching methods for cross sectional data is relevant.^59^ If *v*:1 matching is used and *v*>1, each treated patient will belong to a group of *v*+1 similar individuals, from within the *j*th risk set. The selection of *v*=2 rather than *v*=1, greatly improves precision though it will increase bias if additional matches are not as good.^17^, ^59^ The advantage of increasing *v* quickly diminishes (certainly by 10) and *v* is typically less than 3 in longitudinal matched analyses.^14^, ^15^, ^16^, ^23^

Particularly, when matching without replacement, it may be important to find the optimal matches, across all risk sets, rather than sequentially. So far, a sequential process has been described, in which matching is performed chronologically for each of the observed treatment times, *s*_*j*_. This is analogous to “greedy” matching where the starting point is the first treatment time. However, the “greedy” sequential approach could perform poorly if the quality of matches decays over time. An alternative is to implement optimal matching, simultaneously across all longitudinal risk sets.^14^ Lu (2005) compared simultaneous matching to sequential matching and observed little difference.^15^

### 5.3 Assessment of balance

Whenever matching is used to address imbalance in the covariates, as in Section 5.2.3, the success of the matching strategy should be checked. Methods for the time‐invariant setting have been described elsewhere.^59^, ^60^ Among these methods, standardized differences (absolute difference in covariate means divided by standard deviation) have also been used for longitudinal matched studies.^1^, ^16^ At the initiation of the *j*th pseudo‐experiment, the treated patient has covariates denoted ***Z***_*j*_(*s*_*j*_) and the untreated ***Z***_*i*_(*s*_*j*_). The timing of “baseline” covariates is dictated by the treated patient in each pair. Regarding this as a new baseline, the covariate information over treated patients can be pooled (at their respective baseline) and compared to the untreated patients (at their respective baseline). Following this approach and using the metric of standardized differences, good covariate balance was achieved by longitudinal propensity matching.^1^, ^16^ Balance has also been assessed by hypothesis testing;^15^, ^28^ however, this approach has been discouraged due to its dependence on sample size.^59^

As with traditional treatment comparisons, the propensity‐based approach can be helpful in terms of identifying lack of common support (covariates that are completely different between treatment arms) and imbalance that persists after adjustment. Regression‐based approaches do not warn of poor comparability and model extrapolation,^16^ whereas matching based on sequential strata or a propensity score can reveal cohorts with extremely high propensity to intervention, for whom adequate matches can not be found. These should be excluded during the design phase, without relying on models.^16^ The exclusion of patients near the tails of the propensity distribution is sometimes regarded as a limitation of matching but can also be considered an advantage as these patients are unusual and not representative of the majority of clinical practice.^30^

## 6 ANALYSIS OF THE OUTCOME

The analysis of outcomes is closely integrated with the approach to matching. In Sections 2 and 5, we exemplify models for outcome that have been coupled with matching methods. Here, we focus on the implications to analysis and interpretation at the outcome stage.

### 6.1 Conditional versus marginal treatment effects

Cross‐sectional studies of treatments often differentiate between conditional and marginal effects, which are typically not equal in nonlinear models.^59^ Equations (1), (2), (4), and (5) are all conditional (stratified) on the *j*th pseudo‐experiment and Equations (1), (2), and (5) are additionally conditioned on covariates ***Z***_*i*_(*s*_*j*_), which are essentially baseline values with respect to the *j*th pseudo‐experiment. LM methods have focused on conditional treatment effects,^20^, ^21^, ^23^, ^29^ though some are interested in a marginal treatment effect.^13^, ^26^, ^28^, ^39^ As with cross‐sectional studies of treatment, there may be advantages to further adjustment for confounding by regression on ***Z***_*i*_(*s*_*j*_) and consequently estimating a conditional treatment effect.^59^ These include better adjustment for confounding, greater precision in the effect estimates, and a subject‐specific interpretation of treatment effects.

When the target of inference is a marginal treatment effect, underlying heterogeneity in the treatment effect for individuals will induce different average causal effects for different sub‐populations. This has led to the distinction of parameters such as the average treatment effect (ATE) and average treatment effect among the treated (ATT).^59^ In Section 5.2.3, treated patients form the basis for matching and controls are selected to look like them. With respect to the distribution of risk factors, the matched population resembles treated patients, and the average treatment effect is among the treated, often called the ATT.^1^, ^26^, ^39^ Comparing the matching algorithm in Section 2 to those in Section 5, the latter increasingly match on patient‐specific characteristics, and the matched population will more closely resemble the treated patients. Describing the matched population will help to inform generalizability.

### 6.2 Censoring

**Figure 2:**
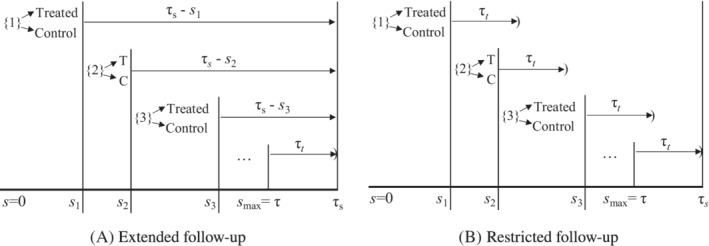
Follow‐up time scales and censoring dates. Time scale, s, represents time since first eligibility for enrollment. Censoring is purely administrative and occurs at a common time point, *C*_*i*_=*τ*_*s*_, for all patients. We are interested in studying outcomes after treatment initiation for a minimum of *τ*_*t*_ years. Treatment initiation times must be limited to *s*∈(0,*τ*). As before, {*j*} represents the set of eligible patients at time *s*_*j*_ satisfying *R*_*i*_(*s*_*j*_)=1, ${ℰ}_{i}(s_{j})=1$, and *S*_*i*_≥*s*_*j*_ for *i*=1,…,*n* patients. Any patients with *S*_*i*_=*s*_*j*_ are treated and the remainder with *S*_*i*_>*s*_*j*_ are controls in the *j*th experiment. A, Extended follow‐up. B, Restricted follow‐up

Observational datasets usually have administrative censoring dates, when the study observation period ends. First, assume that censoring is purely administrative and occurs at a common time point, *C*_*i*_=τ_*s*_, for all patients (Figure 2). This is common in medical claims data when the time scale (*s*) is calendar time and data are available between *s*=0 and *s*=τ_*s*_ years. Even in this simple case, the observed treatment times will not reflect all possible values across the population but a truncated set that depends on the length of follow‐up.^26^ Suppose we are interested in studying outcomes after treatment initiation for a minimum of τ_*t*_ years. The maximum treatment initiation time, such that τ_*t*_ years of outcome follow‐up remain available, is τ_*s*_−τ_*t*_=τ (Figure 2). Treatment initiation times must be limited to *s*∈(0,τ). Inference is restricted to this range and may not generalize beyond.

**Figure 3:**
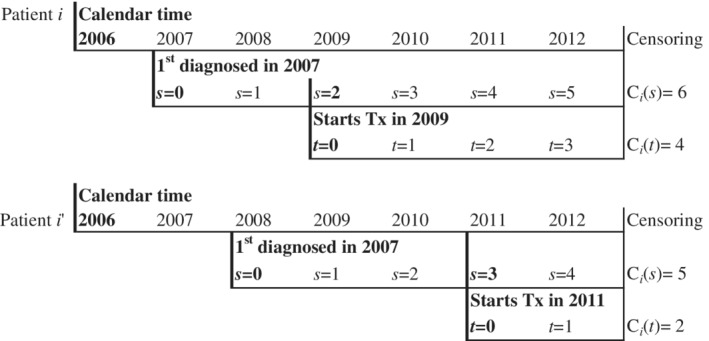
Illustration of two hypothetical patients across three time scales (discrete for simplicity). Available data range across calendar time 2006‐2012. The relevant time scale for matching (*s*) is time since diagnosis. Follow‐up for outcomes (time scale *t*) begins after treatment (Tx) initiation. Censoring time with respect to *s* is *C*_*i*_(*s*) and with respect to *t* is *C*_*i*_(*t*)

Even when censoring is purely administrative, the available follow‐up time for outcomes may vary across individuals with a *maximum* of τ_*s*_. In medical claims data, this scenario will arise when the time scale (*s*) is relative to an initial qualifying event or first diagnosis at which *s*=0. Patients who qualify early (with respect to the range of available data) and/or initiate treatment early (with respect to *s*) will tend to have longer censoring times (Figure 3 and Supplementary Material). The set of observed treatment times depends on the study period (in calendar time) and distribution of first diagnoses. At a minimum, the observed treatment times should be characterized and evaluated for clinical relevance. When the goal is to estimate an average over the treatment initiation times that would have been observed without censoring, a method that explicitly accounts for censoring is needed.^26^ Otherwise, the analysis is implicitly conditional on the observed treatment times and may not generalize to other patterns of care.

In addition to affecting the distribution of observed treatment times, censoring will create differential follow‐up over time‐scale *t* following *s*_*j*_. One option to address this is to censor follow‐up at *s*_*j*_+τ_*t*_ for the *j*th experiment so that all experiments will have the same amount of potential follow‐up, τ_*t*_, see Figure 2B. Again this has the implication of limiting inference to the range *s*∈(0,τ) and *t*∈(0,τ_*t*_). That may or may not be desirable.

To avoid discarding information, one might define $\tau_{\min}$ as the minimum amount of follow‐up required, and $\tau_{\max}$ as the maximum duration of interest. Censoring times *C*_*i*_ will then range between $\tau_{\min}$ and $\tau_{\max}$. Depending on the analysis, this can create a problem of dependent censoring because earlier treatment times *S*_*i*_ will often be correlated with shorter subsequent time to outcome (*Y*_*i*_−*S*_*i*_), and longer times until censoring (*C*_*i*_−*S*_*i*_).^26^ Within matched strata, or within the *j*th experiment, there will be no such correlation when all members of the strata have a common potential follow‐up *C*_*j*_−*S*_*j*_. Equations (1), (2), (4), and (5) are all stratified on the *j*th experiment and may avoid this kind of dependent censoring. However, marginal analyses, such as survival curves based on the Nelson‐Aalen estimator of the cumulative hazard, will be biased. Such analyses require weighting for dependent censoring.^26^ Other causes of loss to follow‐up, such as patient withdrawal from the study, may further justify the use of censoring weights.

### 6.3 Per‐protocol analysis

When the treatment effect of interest is to remain on the initial therapy, a per‐protocol analysis is often conducted. One method of per‐protocol analysis is to censor individuals who cross‐over or switch from their initial treatment status.^4^ This kind of censoring is likely to be informative for the same reasons that treatment initiation was confounded at the start of pseudo‐experiments. If censoring were only related to static characteristics, measured at the beginning of the *j*th experiment ***Z***_*i*_(*s*_*j*_), censoring would be conditionally independent of outcomes given correct model specification of Equations (1), (2), and (5).^23^ However, censoring may also be related to changing values of ***Z***_*i*_(*s*) that are updated during follow‐up for outcomes. Therefore, results will be vulnerable to bias due to dependent censoring. The preceding challenges due to dependent censoring can be addressed by inverse probability of censoring weights (IPCW),^20^, ^21^, ^29^, ^61^ as long as the time‐dependent covariate data are sufficiently rich to capture the difference between patients who are censored earlier versus later.

### 6.4 Effect modification

One of the advantages to LM is the ability to study effect modification of treatment effects by time‐dependent covariates.^20^, ^23^, ^29^ Gran et al (2010) evaluated early and late treatment.^21^ Pseudo‐experiments initiated prior to 12 months were analyzed separately from pseudo‐experiments initiated after 12 months, demonstrating a larger treatment effect with early initiation. Hernan et al (2008) estimated treatment effects of hormone replacement on cardiovascular events separately by baseline age and time since menopause, finding greater harm among older women.^29^ Schaubel et al (2009) detailed a strategy for estimating the effect of a time‐dependent treatment by levels of an internal time‐dependent covariate, in application to effect of liver transplantation versus remaining on the waitlist.^20^ The time‐dependent values of “Model for End‐stage Liver Disease” (MELD) score distinguished patients who would be harmed by transplantation from those who would benefit.

### 6.5 Variance estimation

Early approaches to LM did not allow a patient to be repeated.^14^, ^15^ Once a control was selected for the *j*th pseudo‐experiment, they became ineligible for future experiments (either as a case or control). This has the advantage of ensuring independent observations. In this case, standard model‐based variance estimates will be valid. Recently developed LM methods allow patients to be repeated, potentially as a control for multiple treated patients, or as a control who becomes treated at a later time (Sections 5.2.1 and 5.2.2). They differ in how the resulting correlation is handled in variance estimation. A variety of authors have proposed to use a bootstrap variance estimator.^1^, ^19^, ^20^, ^21^, ^39^ Abadie et al (2006) showed that there is no theoretical justification for a bootstrap variance estimator with matched analyses;^62^ however, recent simulation studies indicate good performance of bootstrap estimators in the context of propensity score matching without replacement.^63^ A sandwich empirical variance estimator has also been used.^22^, ^23^, ^29^ Still others have proposed estimators for specific quantities (survival rates, average hazards) for which an analytic variance can be derived.^26^, ^37^ More research is needed regarding appropriate variance estimation.

## 7 EXAMPLE

We use LM methods to evaluate efficacy of statins for prevention of cardiovascular outcomes in the Framingham Offspring Cohort. The Framingham Offspring Cohort was initiated in 1971, with participants aged 5 to 70 years old, with follow‐up at scheduled examination intervals. Statin initiation was first observed at examination 5 (approximately 1990). At subsequent follow‐up, every 3 to 6 years, updated data were obtained on statin status, covariate information, and outcomes. This corresponds to examinations 5, 6, 7, 8, and 9, last occurring in 2014. Starting at examination 5, there were 5124 patients eligible for this analysis (alive and under follow‐up) and 151, 241, 371, 715, and 340 patients initiated statins at visit 5, 6, 7, 8, and 9, respectively (1818 total statin users). Data collection included major factors known to drive statin selection, including all variables that were ultimately incorporated into lipid guidelines.^12^ Specifically, sex, age, BMI, diabetes, smoking status, history of myocardial infaction (MI), peripheral artery disease (PAD), stroke or atherosclerotic cardiovascular disease (ASCVD), systolic and diastolic blood pressure (SBP and DBP), antihypertensive medications, total cholesterol, HDL cholesterol, triglycerides, and fasting glucose. The outcome of interest is a composite cardiovascular outcome of myocardial infarction, stroke, or cardiovascular death. The dates of outcomes were collected until 2016, 24 years from the first initiation of statins. Data were obtained via the Framingham Heart Study and analyses were approved by Duke Institutional Review Board (IRB Pro00089322). These data are publicly available from the Framingham Heart Study with restrictions. Our data use agreement does not allow us sharing the data directly with any third party. However, a simulated example and corresponding SAS code for all analyses in this section are available upon request.

The target pragmatic trial that we intend to mimic would randomize patients to receive either statin or placebo at each Framingham Offspring examination time, starting with exam 5 and continuing enrollment at subsequent examination periods. All patients who did not previously receive statins will be eligible. Participants assigned to statins will receive a daily dose for the remainder of the study and those assigned to placebo will receive usual care (open‐label). Participants will be followed until the first occurrence of the cardiovascular composite endpoint, death, loss to follow‐up, administrative end of follow‐up in 2016, or 15 years. In alignment with actual clinical trials, this includes a mixture of primary prevention (no prior cardiovascular disease) and secondary prevention (prior cardiovascular disease).^12^ The effects of interest will include both the ITT effect of assignment to statins vs placebo, and per‐protocol effect of starting and adhering to statins vs placebo. Subgroup analysis will be conducted in subgroups defined by: (a) age (<75, ≥75); and (b) primary vs secondary prevention.

To mimic this pragmatic trial, we apply three LM methods: (a) the sequential Cox model of Gran et al (2010);^21^ (b) sequential stratification of Schaubel (2009);^20^ and (c) time‐dependent propensity score matching of Lu (2005).^15^ These methods were selected because they represent three different approaches (as described above) and they provided clear theoretical justification. They also correspond to a number of similar methods (Table 1).

### 7.1 Design

First, we address design considerations that are relevant to all methods, as in Section 4. The time scale for creation of experiments is discrete and corresponds to examinations, that is, *s*=5,6,7,8, or 9. The time scale, *t*, for outcome follow‐up is continuous with outcomes being captured at exact dates. Age is another relevant time scale that is accounted for in the covariate list. In this population, there is no required diagnosis or index event to anchor time‐since‐diagnosis. Such an analysis could have been designed to study statins in patients with prior cardiovascular events. We do not do that here.

It is straightforward to identify new users of statins in the Framingham Offspring Cohort because the cohort began in 1971, before statins were available. No one was taking statins at examination 4 and first initiation was observed at examination 5. No “wash‐out” period is necessary to exclude prevalent users because all initiation is observed. However, one limitation is that study examinations occur only every 3 to 6 years. Therefore, patients who appear to initiate statins at examination 5 (for example) may have been taking statins for a few months or a few years. This is a potentially severe limitation. However, statins are generally considered a long‐term therapy for which benefit may accrue over decades, and relative to a 24‐year follow‐up period, the capture of statin initiation at examinations may be frequent enough.

The current study will define inactive controls such that all patients may be selected as a control if they remain eligible, at risk and do not receive statins. This is meant to align with the target trial (relative to a placebo). A potential risk of this design is that controls may include very healthy patients with virtually no reason to get statins. Validity of LM will depend on the assumption that the 16 covariates described above are sufficient to capture this difference, and there are no unmeasured confounders. Sequential stratification partially mitigates this risk through the use of longitudinal eligibility criteria. Eligibility for statins can be defined by the 2013 ACC guidelines based on age, LDL cholesterol, and other risk factors.^12^ This guideline is generally recognized as defining inclusive eligibility criteria. In our application of sequential stratification, patients are only eligible to enter an experiment if they meet the ACC eligibility criteria. Thus, the target trial is redefined to be a study of eligible patients. Reframing the question to address eligible patients may reduce sensitivity to model assumptions on the measured covariates and potentially even reduce the risk of unmeasured confounding because the model is fit to a more homogeneous population with at least some reason to receive statins. We do not apply this filter for sequential Cox nor time‐dependent propensity score matching because the proposed methods did not.^15^, ^21^

The ITT analysis will estimate the effect of starting statins vs placebo, given that people initiated on placebo can ultimately switch to statins at a later date and vice versa. The per‐protocol analysis will estimate the effect of starting and adhering to statins vs placebo. In the latter analysis, patient follow‐up will be censored at the time of treatment switching. The per‐protocol analysis has a number of advantages. It corresponds to a target trial in which patients could be induced to perfect adherence to statins or placebo. This is a biologically important effect that is not dependent on the behavior of patients in the current sample. The ITT target trial would randomized patients once, at a baseline. Subsequent adherence would be at the discretion of the patient. Outcomes would be compared by randomization not post‐baseline actual treatment status. Both are included for comparison.

### 7.2 Methods

The list of potential confounders was identified a priori by scientific knowledge and informed by the 2013 ACC guidelines.^20^ The 16 variables listed above were assumed to capture all time‐dependent confounding. Values were updated at every visit, with <3*%* missing except for fasting glucose at 8*%*. For this analysis, we required nonmissing covariate data (complete case) to be eligible for an experiment. Covariate information measured at *s*_*j*_, ***Z***(*s*_*j*_), was assumed sufficient to account for confounding in the pseudo‐experiment initiated at *s*_*j*_, and we did not incorporate cumulative measures of longitudinal history $Z_{i}^{*}(s_{j})$. However, lab values were obtained from the prior visit, that is, *s*_*j*−1_, to ensure that labs, such HDL cholesterol, reflect pre‐statin levels and not post‐statin results. Nonlab data were assumed to be more accurate at time *s*_*j*_ because these variables would generally cause statin initiation but not be caused by statins.

Next, we consider decisions related to specific methods. We implemented the sequential Cox model as described in Section 5.2.1. In this analysis, 1667 unique patients initiated statins over five pseudo‐experiments. At every experiment, all available controls were included (11 508 nonunique controls). The model defined in Equation (1) was fit with a Cox proportional hazard model, stratified on examination visit (ie, pseudo‐experiment) conditional on information known at the start of the experiment ***Z***(*s*_*j*_). All decisions follow the original publication with the exception of our model for censoring. We use a Cox proportional hazards model for censoring. This was done for convenience and comparability with other methods. All methods use the same model for censoring weights (where applicable).

Sequential stratification, Section 5.2.2, begins with the designation of strata variables. We defined strata based on the five examination periods, history of ASCVD (ie, primary vs secondary prevention), age within 5 years, and Framingham Risk Score within 1*%*. The Framingham Risk Score was developed separately to predict risk of cardiovascular events within 10 years. Combinations of these variables defined 4000 potential strata (800 per examination). To enter each pseudo‐experiment, statin treated patients and their controls were required to be eligible according to the ACC 2013 statin guidelines and then grouped according to their derived strata. 440 statin‐treated patients were excluded because no controls existed within their strata. A total of 657 strata included 1227 unique statin‐treated patients and 4490 nonunique controls. The model defined in Equation (2) was fit with a Cox proportional hazard model, stratified on the derived strata variable, and conditional on remaining covariates known at the start of the experiment ***Z***(*s*_*j*_).

Finally, we apply time‐dependent propensity score matching with the corresponding outcome model in Equation (4), Section 5.2.3. In the current data, it is likely that the time‐dependent propensity to receive statins is changing over 24‐year follow‐up and cannot be modeled by a simple proportional hazards model. To address this, we adapted the model proposed in Equation (3) to accommodate examination‐specific propensities. We conducted 1:1 matching at each examination, starting with exam 5, and excluded patients from future risk sets once they had been matched (per Lu 2005). We applied a caliper of 0.25 times the standard deviation of the linear propensity score predictor. Five hundred and nineteen statin‐treated patients were excluded for failure to find a match. The matched sample included 1148 unique statin‐treated patients and 1148 unique control patients. The final Cox proportional hazards model for outcome was stratified on matched pair, and not conditioned on any covariates (Equation (4)).

**Figure 4:**
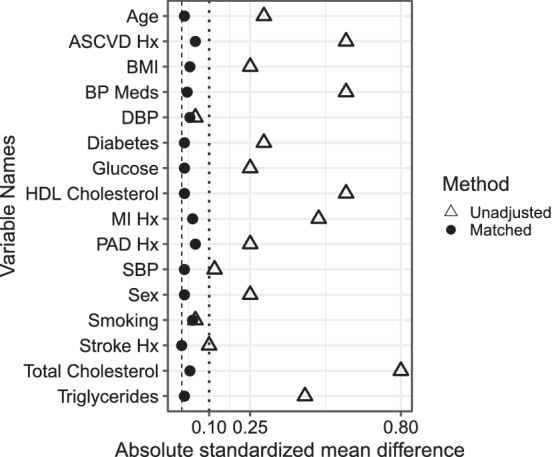
Balance check at examination 7. Absolute standardized mean differences are the difference in covariate means between two groups (treated vs untreated) divided by the standard deviation of the same covariate. Unadjusted: patients who initiate statins are compared to eligible controls at examination 7. Matched: after matching on a time‐dependent propensity score, patients who initiate statins and their matched controls at examination 7 are compared

We note a number of differences between these methods. First, both Gran et al (2010) and Schuabel et al (2009) allow control patient to be reused and to become treated patients in another experiment. In contrast, Lu (2005) matches without replacement, and a number of treated patients fail to find a good match. If we attempted to find more than 1 control per treated patient (1:v matching), even more controls would be “used up” and unavailable for future experiments. The approach could easily be adapted to allow matching with replacement. However, with this change, it would become very similar to sequential stratification. An advantage to propensity score matching on a time‐dependent covariate is that balance can be evaluated after matching. For example, in Figure 4, standardized mean differences in covariates between statin patients and matched controls are nearly 0 at examination 7. Results are other time points were similar.

### 7.3 Analysis

All of the methods considered here estimate conditional treatment effects; the outcome model is conditional on “baseline” information (where baseline is defined with respect to the pseudo‐experiment). The sequential Cox approach estimates a treatment effect conditional on ***Z***(*s*_*j*_), whereas sequential stratification has a within‐strata interpretation that is also conditional on ***Z***(*s*_*j*_), and time‐dependent propensity score matching yields a paired data interpretation (among two people with the same propensity score).

For each method, we estimate an ITT effect (without censoring at treatment switching), and a per‐protocol effect where patients are censored if they deviate from the initial treatment status. In the per‐protocol analyses, approximately 10% of patients initially treated with statins were censored for stopping during follow‐up, and 30% of controls were censored during follow‐up. Both Gran et al (2010) and Schuabel et al (2009) defined IPCW to account for the informative nature of this censoring. That is, censoring due to switching during follow‐up is likely explained by the same time‐dependent factors that cause treatment decisions in the initial assignment. We apply these IPCW weights for both methods.

Finally, LM methods facilitate the study of effect modification by time‐varying factors. Important subgroups for statins were described above (based on age and prior ASCVD). These subgroups are investigated for the methods sequential Cox and sequential stratification by splitting patients within each experiment according to their subgroup. For sequential stratification, both age and prior ASCVD are part of the original stratification, so we are partitioning strata into the corresponding subgroups. This approach does not work for the method of Lu (2005), at least as originally proposed. Creating subgroups based on age or prior ASCVD would split propensity‐matched pairs because the pairing was not done within these subgroups.

For all methods, we account for correlation induced by repeated patients and/or propensity estimation via a robust sandwich variance estimator.

### 7.4 Results

**Figure 5:**
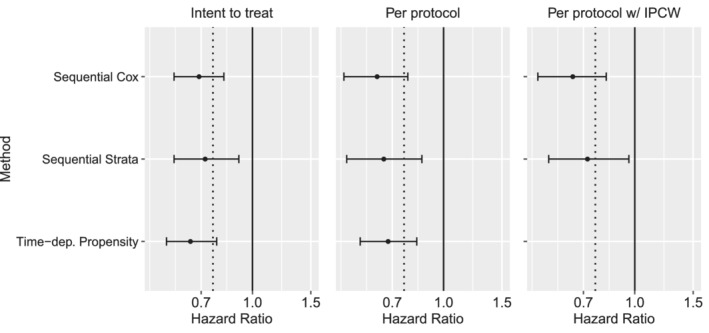
Hazar d ratios for the treatment effect of statins on cardiovascular outcomes over 15 years follow‐up

The estimated treatment effect of statins on the composite cardiovascular outcome is displayed in Figure 5 along with 95*%* confidence intervals. The three LM methods provide similar point estimates and confidence intervals, in both ITT and per‐protocol analyses. The consistency of ITT and per protocol analyses is likely attributable to the fact that switching from the initial treatment assignment was relatively uncommon. The estimated hazard ratios are close to the benchmark of 0.76 observed in a meta‐analysis of clinical trials.

**Figure 6:**
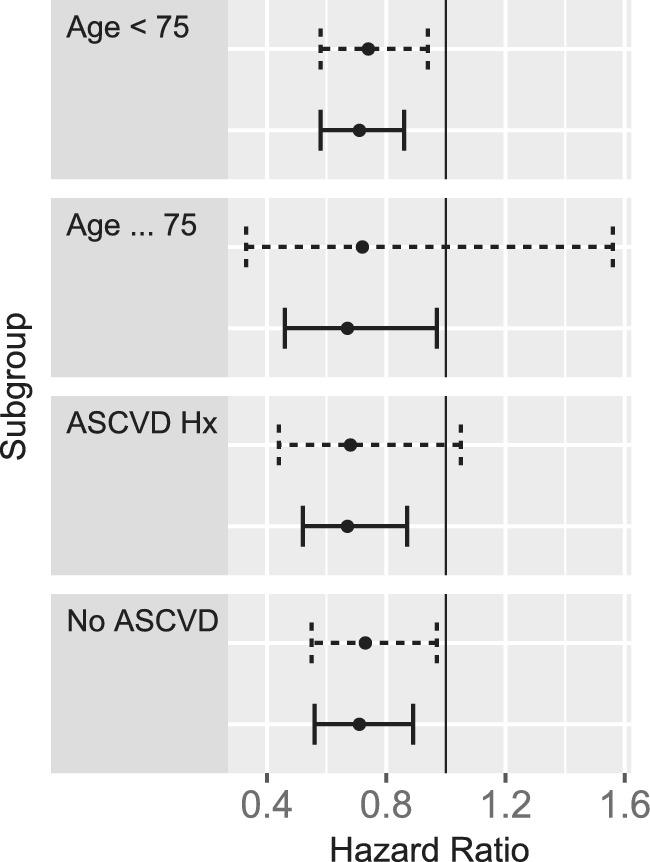
Subgroup analysis of the ITT effect of statins on cardiovascular outcomes over 15 years follow‐up. Sequential stratification (dashed line) and Sequential Cox model (solid line)

Despite similar results, a few differences between these methods are worth noting. First, the sequential Cox model includes 1667 unique statin treated patients and 11 508 controls (not unique). Sequential stratification includes 1227 treated and 4490 controls (not unique). The difference, compared to sequential Cox, is that some strata have no comparators (either all statin treated or all controls) and are thus dropped from analysis. Time‐dependent propensity score matching includes 1148 unique statin treated patients and unique controls. By including all possible statin treated patients, the sequential Cox model achieves slightly narrower confidence intervals (Figure 5). However, some of these statin treated patients were not similar to any controls. By relying on a parametric model to span that difference, this method may have increased risk of bias. Sequential stratification would seem to be a compromise between the other two methods, with some factors used as strata and others in modeling. However, we used a narrow definition of strata, requiring very close agreement in Framingham risk (within 1*%*), history of cardiovascular disease (identical), and age (within 5 years). These narrow strata were intended to ensure comparability of patients within strata, but resulted in 440 statin treated patients being excluded. A broader definition of strata would have preserved more patients and improved precision at the risk of increased model sensitivity. The choice of strata introduces some subjectivity. Finally, time‐dependent propensity matching included the fewest patients overall but has similar results. This result is analogous to cross‐sectional data where matching can reduce variance despite the smaller sample size. Adaptations of time‐dependent propensity matching could improve precision by allowing for repeated patients over time. The results were consistent across subgroups of age and ASCVD (Figure 6).

## 8 DISCUSSION

LM methods are particularly relevant to studying the effects of treatments in large observational studies, recently referred to as “big data.” As randomized clinical trials should remain the preferred choice for establishing causality, there are many cases where clinical trials are not feasible or ethical. When observational comparisons are necessary, it is possible to emulate the attributes of a clinical trials beyond randomization.^33^, ^45^ Big data are often rich with longitudinal information, but require a principled approach to study design. LM methods facilitate this by supporting a new user design with clear eligibility criteria, longitudinal covariate ascertainment, and relevant follow‐up for outcomes, among other attributes.

When treatments vary over longitudinal follow‐up, the choice of methodology should be grounded in the clinical question. If the target trial would enroll patients longitudinally, and randomize them to one or more treatments, LM may emulate this design from observational data. As we have seen, these methods easily incorporate time‐varying eligibility, allowing for effect modification by time‐varying covariates (known at baseline to an experiment) and for effect modification by time (*s*) itself. Finally, the results of LM may be transparent to a scientific audience, who can understand how a target trial is being emulated and even assess covariate balance. Some limitations are also transparent, in which failure to find good matches, or obtain a representative sample of treatment initiation times, will be visible.

Marginal structural models (MSMs) are also used in this setting and may yield practically similar results.^23^ However, MSMs are designed to answer a different question, where follow‐up for outcomes begins at a different baseline, *s*=0 as opposed to *s*=*s*_*j*_, and the causal question pertains to time‐varying treatment strategies. Robins et al (2007) described this problem and introduced a g‐computation formula to estimate causal effects in the presence of time‐varying treatment, where confounders are subsequently impacted by treatment.^9^ He and colleagues contributed statistical methodology including MSMs^64^ and g‐estimation of structural nested models (SNMs),^65^ which have been reviewed in a recent tutorial.^10^ Unsurprisingly, methods that accommodate a complicated question have limitations. The interpretation and assumptions underlying these methods are hard to communicate to a nonstatistical audience. MSMs, for example, involve arbitrary parametric assumptions unless scientific knowledge about the structure of causal relationships is accurate^10^, ^56^, ^66^ and produce unstable estimates in some circumstances.^21^, ^67^, ^68^ When time‐varying treatment strategies are of interest, it is necessary to work through these challenges. Otherwise, alternatives like LM have value.

Many open questions remain. More work is needed to clarify the relative advantages of different LM methods. While these approaches have often been compared to naive methods or entirely different approaches (like MSM), few direct comparisons exist. Simulation studies comparing the relative efficiency, variance estimation, and model sensitivity are lacking. Only a few approaches have been connected with counterfactual outcomes framework for defining the target of interest.^26^, ^39^ In that framework, Li et al (2014) identified potential biases if standard analyses are applied to the matched sample.^26^ Those topics are discussed in Section 6.2 and can generally be handled by inverse weighting for censoring. However, other authors have suggested that once matches have been created across longitudinal cohorts, the analysis can proceed without further adjustment.^13^ The latter is very appealing and suggests the use of standard methods for matched data. More research is needed to clarify the advantages and disadvantages of alternative analytic approaches.

## CONFLICT OF INTEREST

The authors declare no potential conflict of interests.

## AUTHOR CONTRIBUTIONS

L.E.T. conceived and drafted the manuscript. D.E.S. provided critical commentary and revision. D.W. prepared the Framingham data, conducted analysis, and provided comments. S.Y. conducted analysis and provided comments.

## Supporting information

Data S1: Supporting information
