# Supplementary material for: Matching with time‐dependent treatments: A review and look forward
Source: Stat Med. 2020 Apr 3;39(17):2350–70. doi: 10.1002/sim.8533 (PMC7384144; doi:10.1002/sim.8533)
Supplement: Supplementary file 1 — Data S1: Supporting information [file SIM-39-2350-s001.pdf]

Supplementary Material for Matching with Time-dependent Treatments; A Review and Look Forward

February 23, 2020

1 Matching Schema

Figure 1: Longitudinal matching schema similar to Gran et al. (2010); Design 1

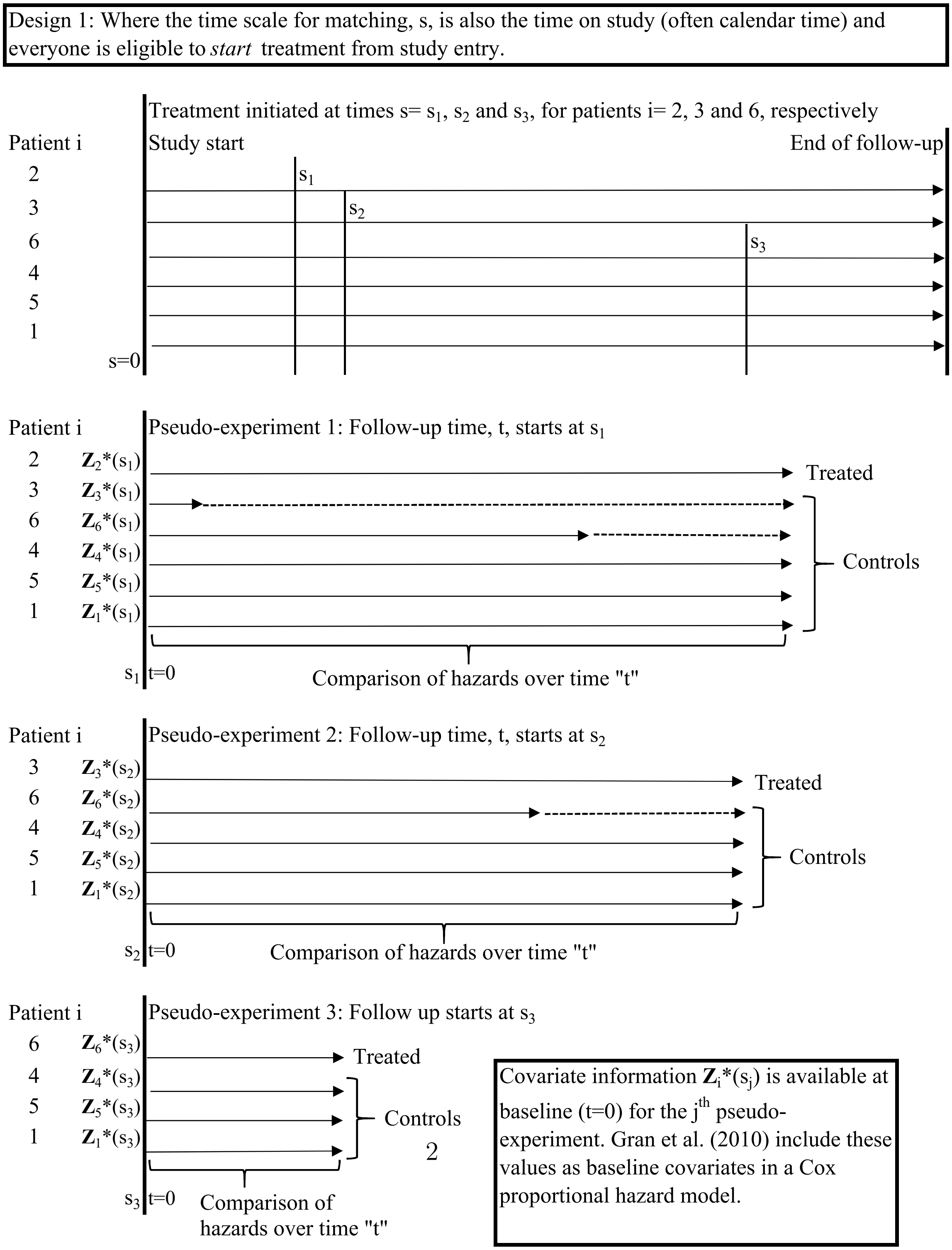

Figure 2: Longitudinal matching schema similar to Gran et al. (2010); Design 2

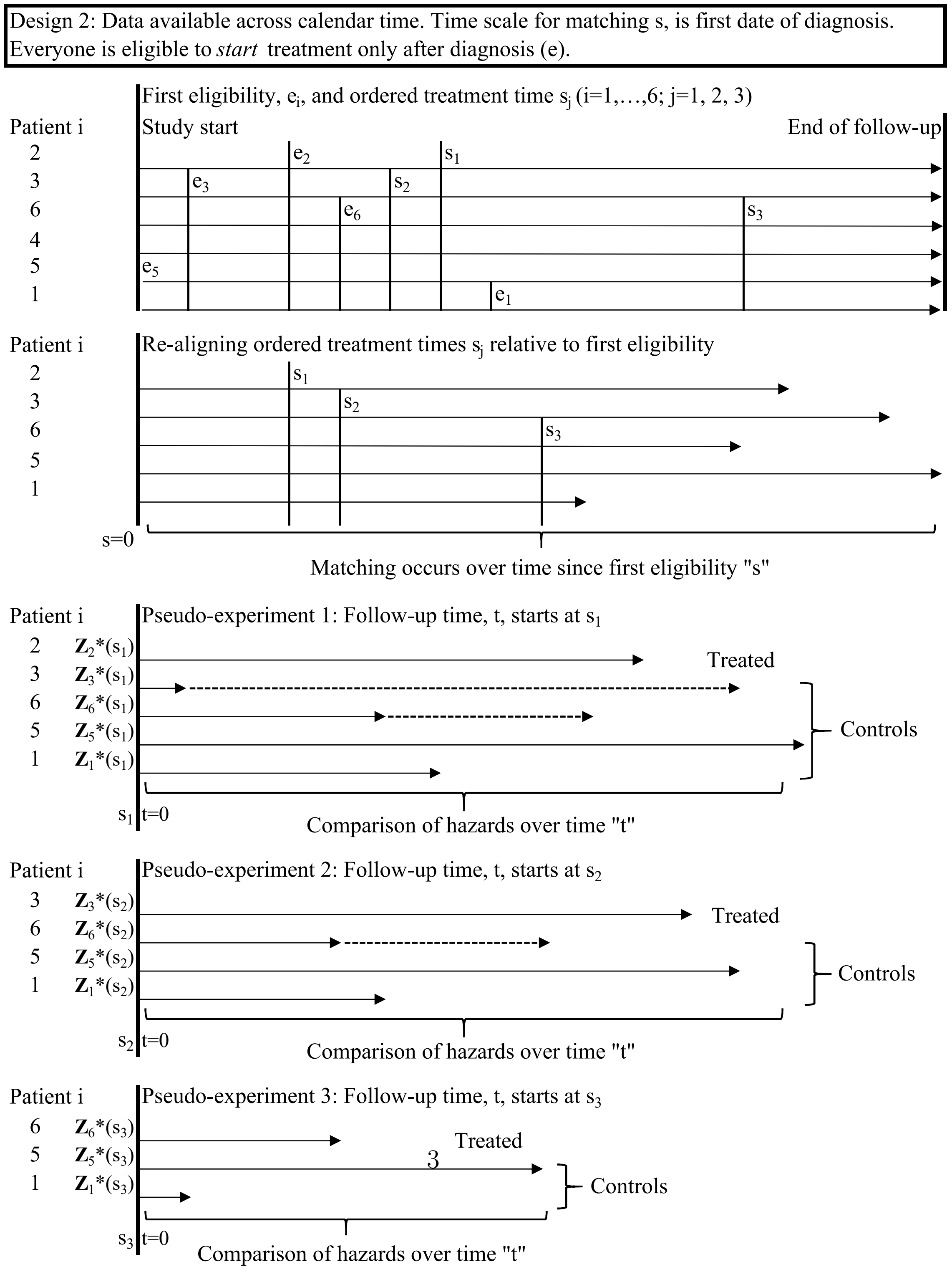

Figure 3: Longitudinal matching schema similar to Lu (2005); Design 1

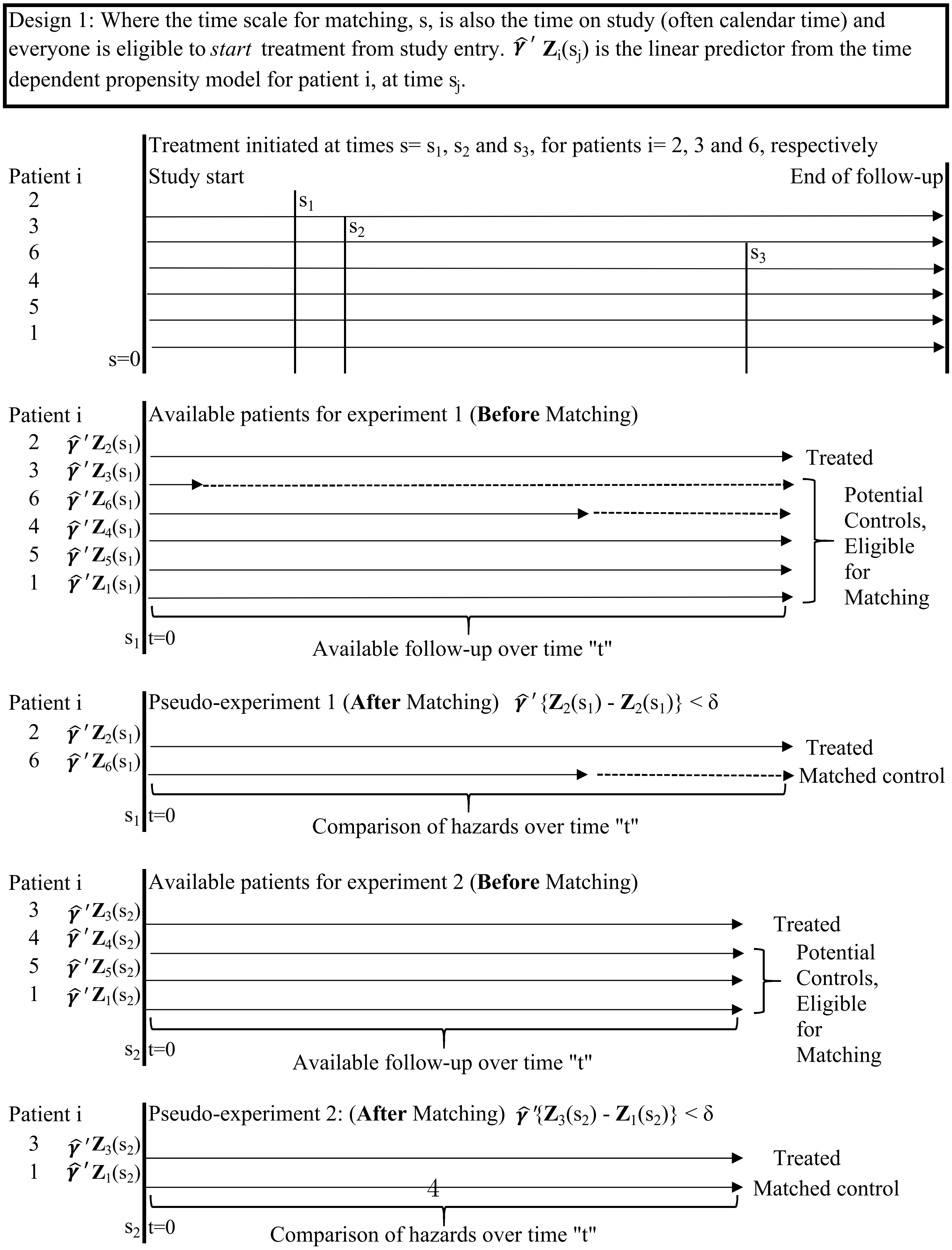

Figure 4: Longitudinal matching schema similar to Lu (2005); Design 2

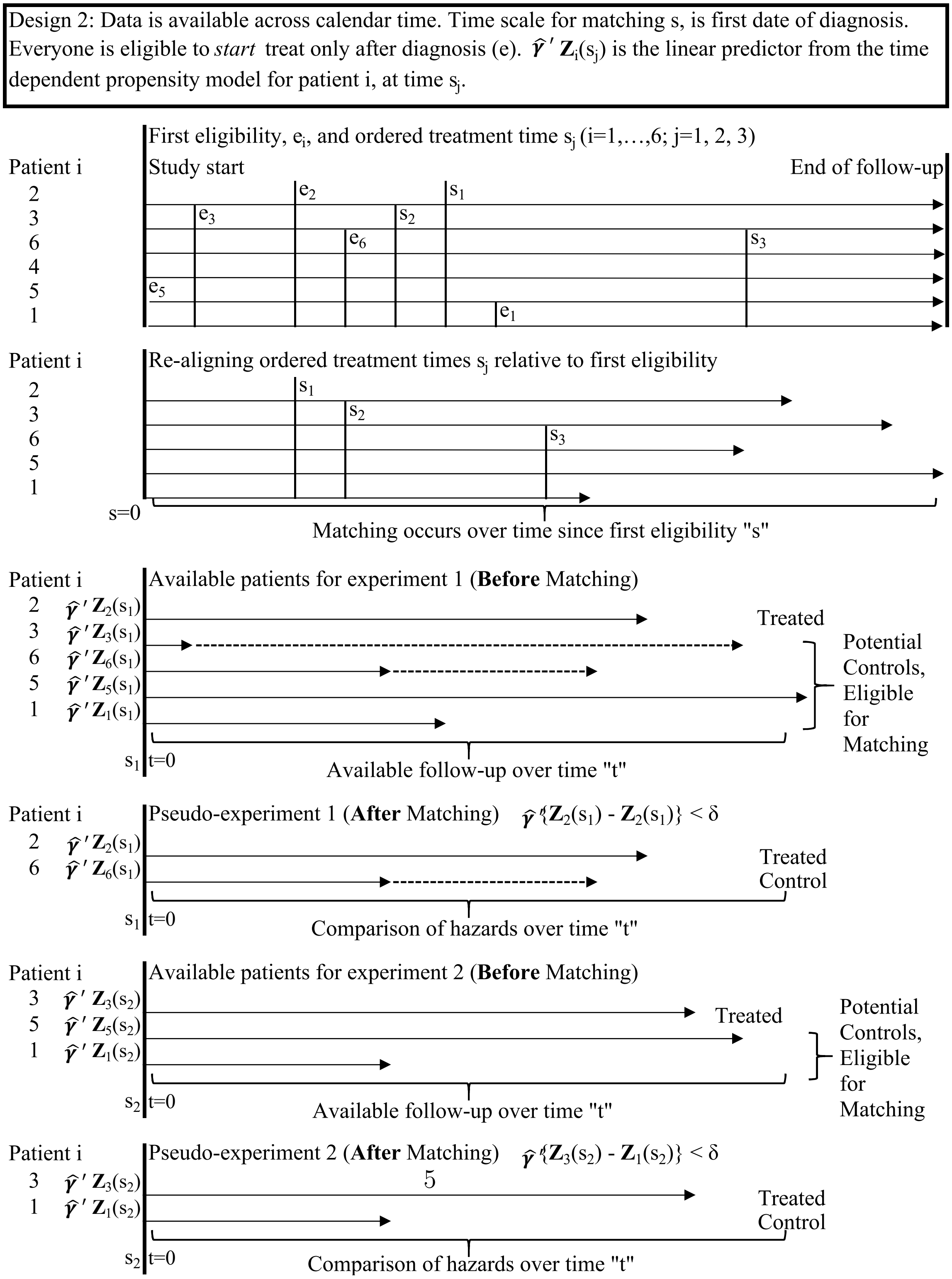

Figure 5: Longitudinal matching schema similar to Schaubel et al. (2006); Design 1

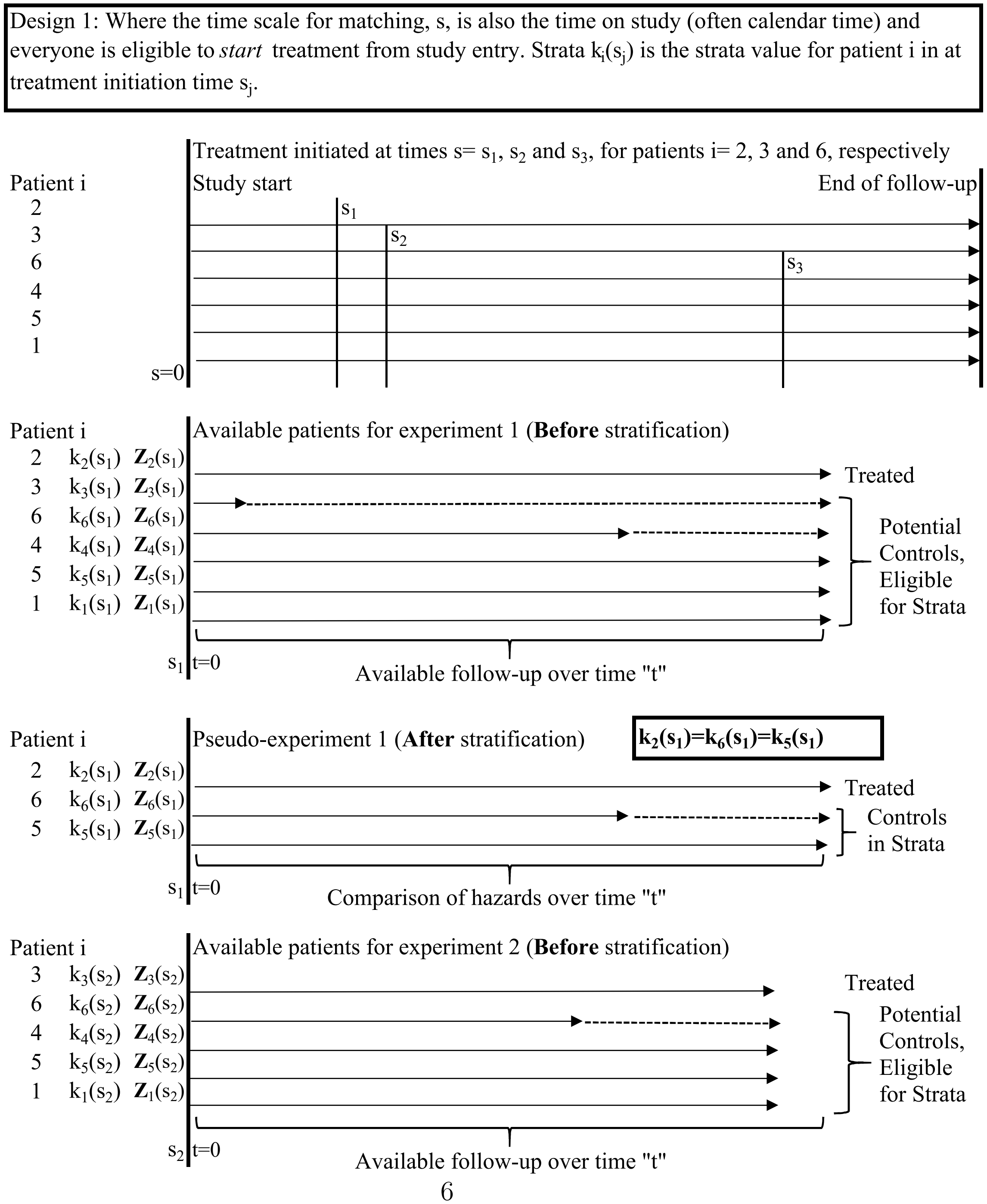

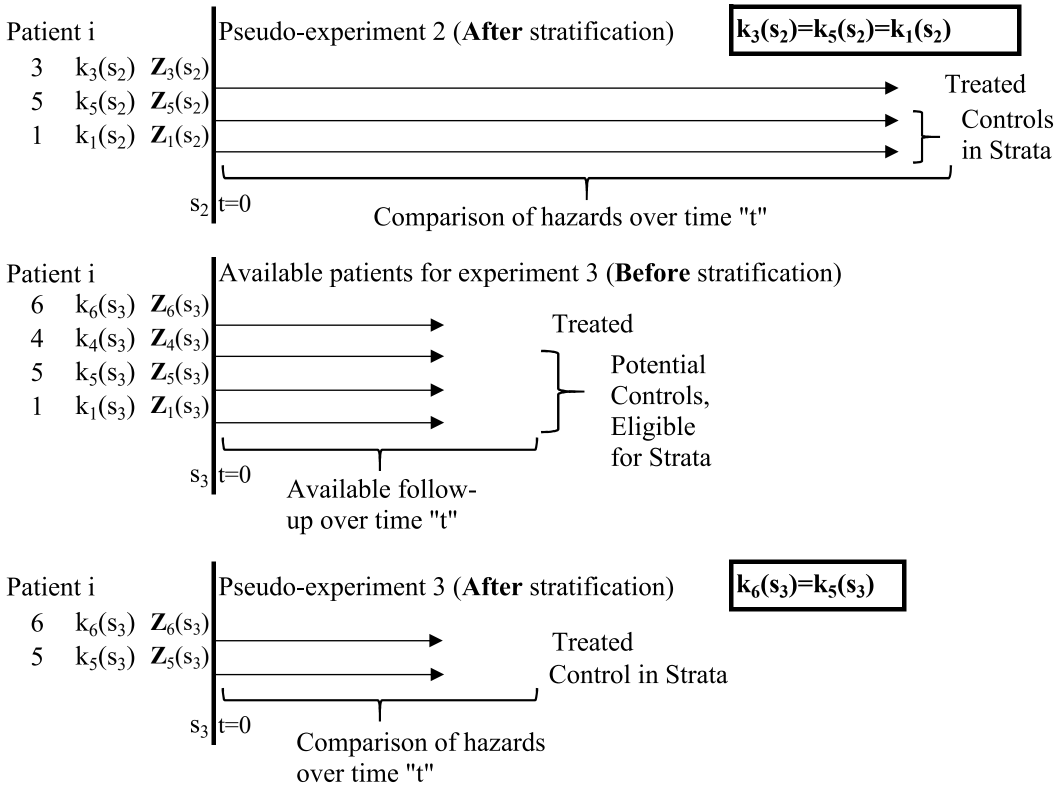

Patient i
3 k3(s2) Z3(s2)
5 k5(s2) Z5(s2)
1 k1(s2) Z1(s2)
Pseudo-experiment 2 (After stratification)
k3(s2)=k5(s2)=k1(s2)
Treated
Controls in Strata
s2 t=0
Comparison of hazards over time "t"
Patient i
6 k6(s3) Z6(s3)
4 k4(s3) Z4(s3)
5 k5(s3) Z5(s3)
1 k1(s3) Z1(s3)
Available patients for experiment 3 (Before stratification)
Treated
Potential Controls, Eligible for Strata
s3 t=0
Available follow-up over time "t"
Patient i
6 k6(s3) Z6(s3)
5 k5(s3) Z5(s3)
Pseudo-experiment 3 (After stratification)
k6(s3)=k5(s3)
Treated
Control in Strata
s3 t=0
Comparison of hazards over time "t"

Figure 6: Longitudinal matching schema similar to Schaubel et al. (2006); Design 2

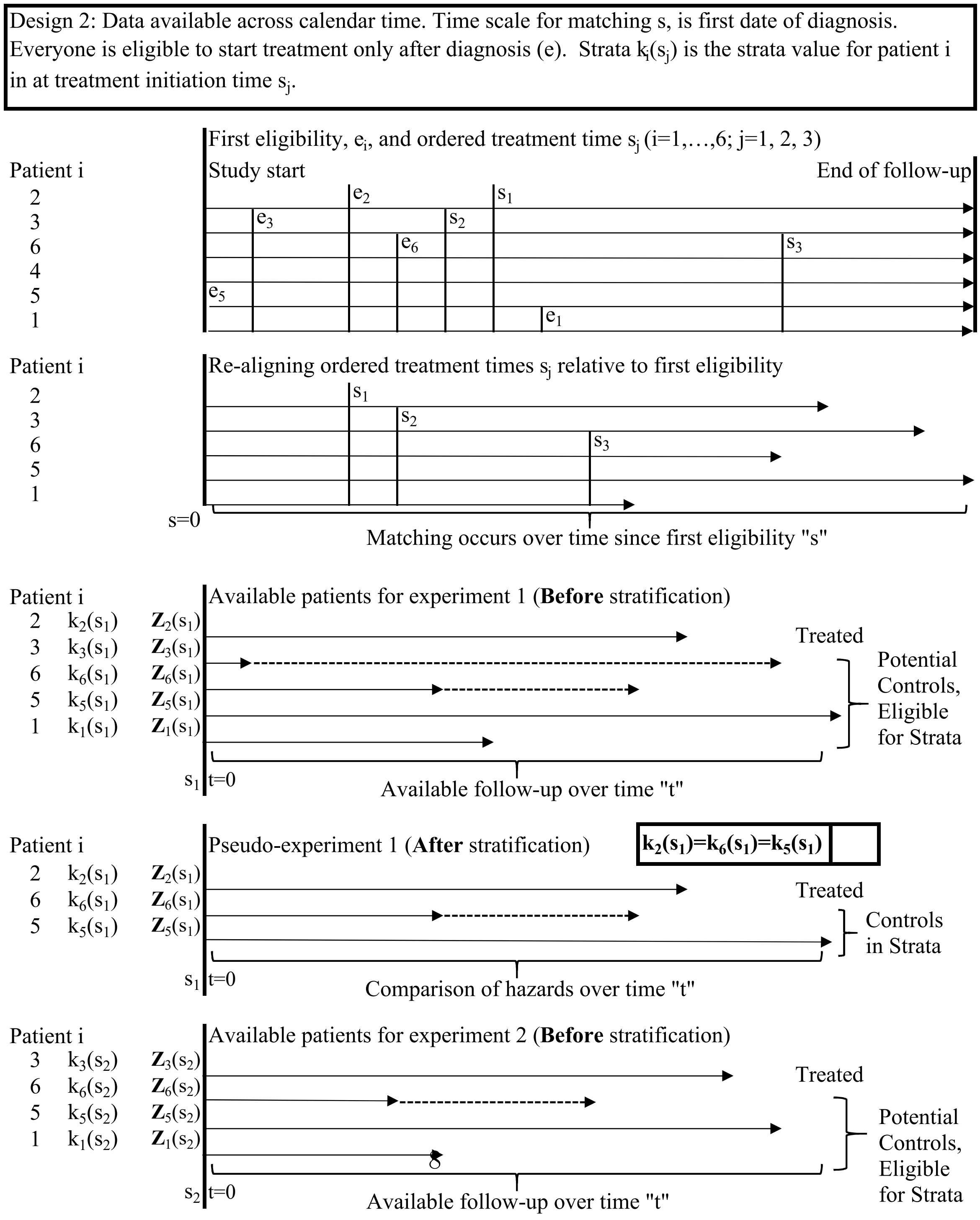

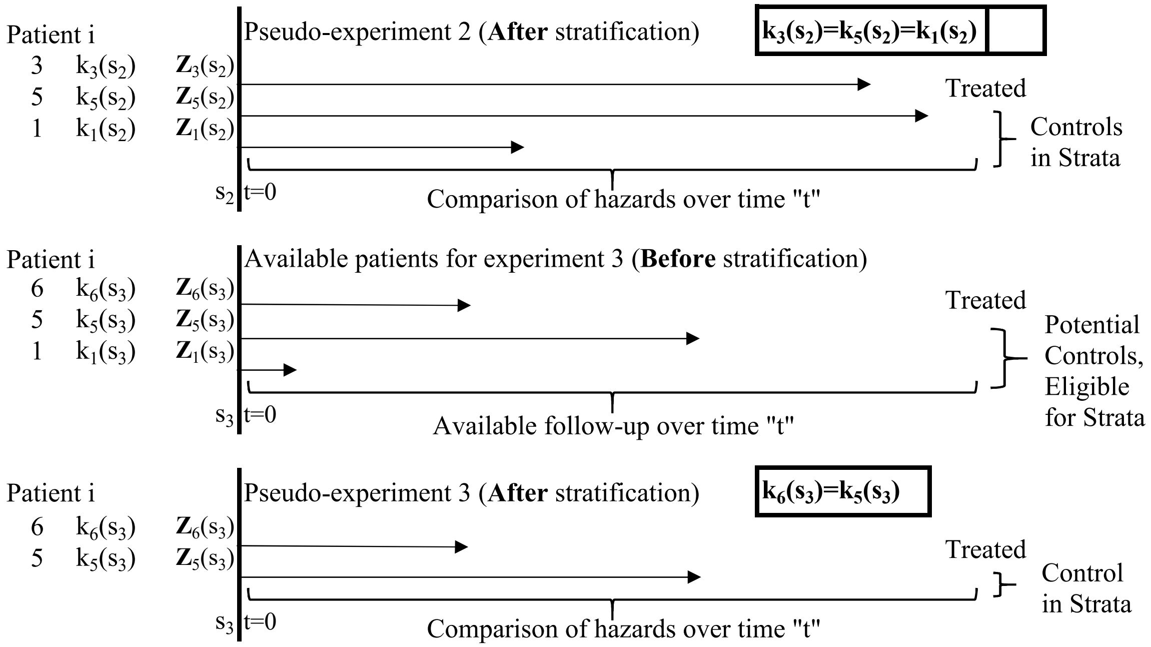
Patient i
3 k3(s2) Z3(s2)
5 k5(s2) Z5(s2)
1 k1(s2) Z1(s2)
Pseudo-experiment 2 (After stratification)
k3(s2)=k5(s2)=k1(s2)
Treated
Controls in Strata
s2 t=0
Comparison of hazards over time "t"
Patient i
6 k6(s3) Z6(s3)
5 k5(s3) Z5(s3)
1 k1(s3) Z1(s3)
Available patients for experiment 3 (Before stratification)
Treated
Potential Controls, Eligible for Strata
s3 t=0
Available follow-up over time "t"
Patient i
6 k6(s3) Z6(s3)
5 k5(s3) Z5(s3)
Pseudo-experiment 3 (After stratification)
k6(s3)=k5(s3)
Treated
Control in Strata
s3 t=0
Comparison of hazards over time "t"

2 Alternative notation for sequential stratification

The combinations of age (70 levels) and state (50 levels) yield 3500 strata, potentially observed at each of j experiments, $k(s_j) = 1, \ldots 3500$. Let $k_i(s_j)$ denote the stratum membership at the time of the jth experiment for the ith patient, and $\boldsymbol{Z}_i(s_j)$ includes the remaining covariates. All patients are entered into the jth experiment if they remain at risk, $R_i(s_j) = 1$, eligible, $\mathcal{E}_i(s_j) = 1$, they were not treated in a previous experiment, $S_i \geq s_j$. These patients are grouped into common strata such that $k_i(s_j) = k_{i'}(s_j) = k(s_j)$ for two unique patients with index i and i' in the jth experiment. The controls are those with $S_i > s_j$ and cases have $S_i = s_j$. Thus treated patients are matched exactly to all eligible controls based on the combinations of covariates used to define strata.

For a time-to-event outcome, D_i, the model for the hazard is:

$$\lambda_{i,k(s_j)}(t; k(s_j)|\boldsymbol{\theta}, \beta) \;=\; \lambda_{0,k(s_j)}(t; k(s_j)) \exp\{\boldsymbol{\theta}' \boldsymbol{Z}_i(s_j) + \beta I(S_i = s_j)\}, \quad (1)$$

where $\lambda_{0,k(s_j)}(t; k(s_j))$ is the baseline hazard at t time units following time s_j for the matched stratum $k(s_j)$. Compared to Eq. 5.1, this applies to a more limited subgroup of patients, specifically those with $k_i(s_j)$ equal to $k(s_j)$ and $\lambda_{0,k(s_j)}(t; k(s_j)) = \lim_{\delta \to 0} \delta^{-1} P\{t \leq D_i < t + \delta | D_i \geq t, S_i > s_j, R_i(s_j) = 1, \mathcal{E}_i(s_j) = 1, \boldsymbol{Z}_i(s_j) = 0, k_i(s_j) = k(s_j)\}$. The information across n_S pseudo-experiments are combined to estimate an overall treatment effect by fitting a stratified Cox model, stratifying on the combination of j experiments and all observed values of $k(s_j)$.
